# Design, Synthesis and the Biological Evaluation of New 1,3-Thiazolidine-4-ones Based on the 4-Amino-2,3-dimethyl-1-phenyl-3-pyrazolin-5-one Scaffold

**DOI:** 10.3390/molecules190913824

**Published:** 2014-09-04

**Authors:** Maria Apotrosoaei, Ioana Mirela Vasincu, Maria Dragan, Frédéric Buron, Sylvain Routier, Lenuta Profire

**Affiliations:** 1 Department of Pharmaceutical Chemistry, Faculty of Pharmacy, University of Medicine and Pharmacy “Grigore T. Popa”, 16 University Street, Iasi 700115, Romania; E-Mails: mariasutu@yahoo.com (M.A.); ioanageangalau@yahoo.com (I.M.V.); mwolszleger@yahoo.com (M.D.); 2 Institute of Organic and Analytical Chemistry, University of Orléans, Orléans 45076, Cedex 2, France; E-Mail: frederic.buron@univ-orleans.fr

**Keywords:** 4-aminophenazone, thiazolidine-4-one, synthesis, spectroscopic methods, antioxidant effects

## Abstract

New thiazolidine-4-one derivatives based on the 4-aminophenazone (4-amino-2,3-dimethyl-1-phenyl-3-pyrazolin-5-one) scaffold have been synthesized as potential anti-inflammatory drugs. The pyrazoline derivatives are known especially for their antipyretic, analgesic and anti-inflammatory effects, but recently there were synthesized new compounds with important antioxidant, antiproliferative, anticancer and antidiabetic activities. The beneficial effects of these compounds are explained by nonselective inhibition of cyclooxygenase izoenzymes, but also by their potential scavenging ability for reactive oxygen and nitrogen species. The structure of the new compounds was proved using spectroscopic methods (FR-IR, ^1^H-NMR, ^13^C-NMR, MS). The *in vitro* antioxidant potential of the synthesized compounds was evaluated according to the ferric reducing antioxidant power, phosphomolydenum reducing antioxidant power, DPPH and ABTS radical scavenging assays. The chemical modulation of 4-aminophenazone (**6**) through linkage to thiazolidine-propanoic acid derivatives **5a**–**l** led to improved antioxidant potential, all derivatives **7a**–**l** being more active than phenazone. The most active compounds are the derivatives **7e**, and **7k**, which showed the higher antioxidant effect depending on the antioxidant assay considered.

## 1. Introduction

The pyrazolin-5-one scaffold occupies an important place in the nonsteroidal anti-inflammatory drug (NSAID) class, having drawn considerable attention from researchers due to its interesting biological activities. Since the first pyrazolin-5-one derivative, named antipyrine, as synthesized by Ludwig Knorr in 1883, many pyrazoles, pyrazolin-5-ones and pyrazolidine-3,5-diones have been developed [1,2,3]. The interesting antiinflammatory, analgesic, antipyretic [2], antirheumatic [4], antidiabetic [5], antioxidant [6], anticancer, antiproliferative [7,8], antifungal and antimicrobial effects [9] of these compounds have been reported. Some of them, such as phenylbutazone, dipyrone, propyfenazone, ramifenazone, suxibuzone, are important drugs with clinical use in the treatment of fever, arthritis, musculoskeletal and joint disorders [10]. 

Phenazone or antipyrine (2,3-dimethyl-1-phenyl-3-pyrazolin-5-one) is a well-known compound for its analgesic and antipyretic effecs, while its 4-amino derivative (4-amino-2,3-dimethyl-1-phenyl-3-pyrazolin-5-one) also has anti-inflammatory effects [11]. Concerning the mechanism of action, the pyrazolin-5-one derivatives are known as nonselective COX isoenzyme inhibitors which inhibit platelet thromboxane and prostanoid synthesis [11]. The biological effects of these compounds have also been attributed to their scavenging ability against reactive species. It is proven that phenazone has good scavenging ability for reactive oxygen species (ROS), especially for hydroxyl radicals, while 4-aminophenazone evidenced a higher scavenging ability for oxygen (peroxyl, hydroxyl, superoxide radicals) and also for nitrogen reactive species (nitric oxide, peroxynitrite) [12]. However, apart from the beneficial effects of pyrazoline derivatives, therapy with these compounds has been associated with several side effects. The most frequently reported side effects are skin rashes, gastrointestinal irritation, cardiovascular (agranulocytosis, blood dyscrasias) problems and renal injury [13].

In order to improve the safety profile and pharmacological effects of the classical anti-inflammatory drugs, in the last years research was been focused on chemical modulation of their structure with different heterocyclic systems such as thiazoles, thiadiazoles, triazoles and pyrimidines [14,15,16,17]. Among them the thiazolidine moiety seems to be an interesting system due to its own biological effects. Compounds with thiazolidine structures have been reported as anti-inflammatory and analgesic [18], antitubercular [19], antimicrobial and antifungal [20], antiviral (especially as anti-HIV agents [21]), anticancer, antioxidants [18], anticonvulsants [22] and antidiabetic agents [22,23]. In the present work, we report the synthesis, structural characterization and antioxidant activity of some new thiazolidine-4-one derivatives that contain a pyrazoline-5-one moiety.

## 2. Results and Discussion

### 2.1. Chemistry

**Scheme 1:**
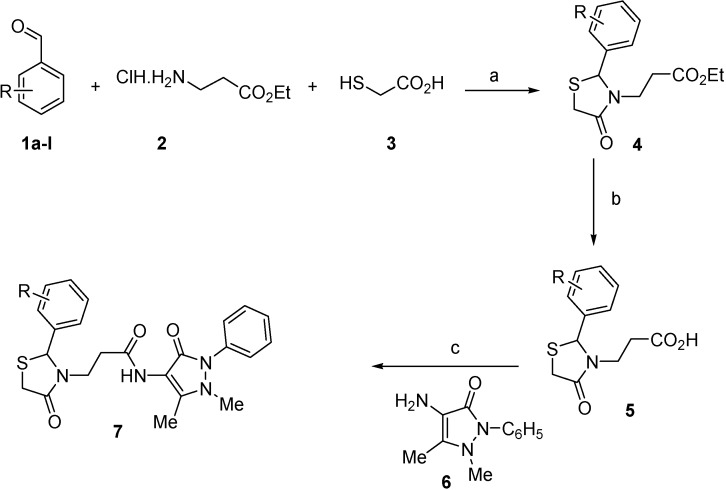
Synthesis of compounds **7**.

Synthesis of 1,3-thiazolidine-4-one derivatives **7a**–**l** was carried out in several steps as is summarized in Scheme 1 and Table 1. Firstly, new ethyl 3-(2-aryl-4-oxo-thiazolidin-3-yl)-propionates **4a**–**l** were obtained in moderate to good yields using a one-pot condensation/cyclization reactions between substituted aromatic aldehydes **1a**–**l**, ethyl 3-aminopropionate hydrochloride (**2**) and mercaptoacetic acid (**3**). In the second step, the treatment of compounds **4** with KOH led to the corresponding acid derivatives **5a**–**l**.

*Reagents and conditions*: (**a**) DIPEA, toluene, reflux 24–36 h; (**b**) KOH 1 M, EtOH/THF (1/1), r.t., 6–10 h then HCl 1 M; (**c**) HOBt, ECDI HCl, DCM; r.t., 8–12 h.

**Table 1:** Synthesis of derivatives **4**, **5** and **7**.

Entry	Product 4	No., Yield	Product 5	No., Yield	Product 7	No., Yield
1	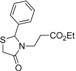	**4a**, 56%	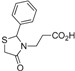	**5a**, 56%	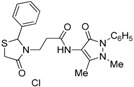	**7a**, 52%
2	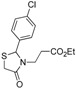	**4b**, 46%	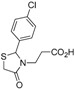	**5b**, 63%	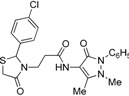	**7b**, **74%**
3	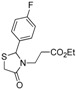	**4c**, 42%	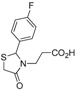	**5c**, 70%	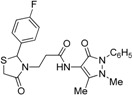	**7c**, **57%**
4	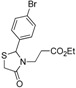	**4d**, 76%	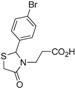	**6d**, **75%**	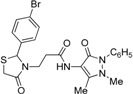	**7d**, **92%**
5	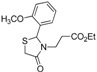	**4e**, 82%	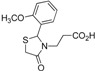	**6e**, **79**%	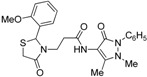	**7e**, **60**%
6	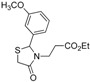	**4f**, 72%	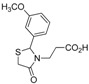	**6f**, **73**%	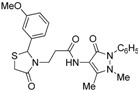	**7f**, **75**%
7	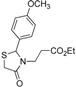	**4g**, 71%	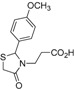	**6g**, **83**%	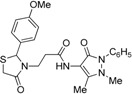	**7g**, **86**%
8	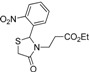	**4h**, 95%	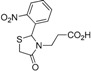	**6h**, **50**%	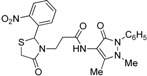	**7h**, **62**%
9	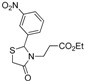	**4i**, 36%	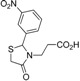	**6i**, **55**%	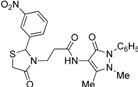	**7i**, **77**%
10	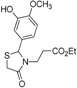	**4j**, 56%	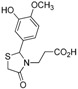	**6j**, 75%	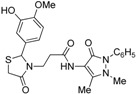	**7j**, **62**%
11	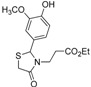	**4k**, 34%	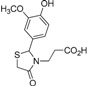	**6k**, 60%	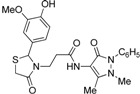	**7k**, **67**%
12	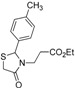	**4l**, 77%	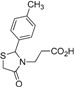	**6l**, 71%	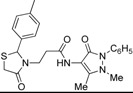	**7l**, 66%

In the last step, compounds **5a**–**l** were reacted with 4-amino-phenazone (**6**) in presence of *N*-(3-dimethylaminopropyl)-*N′*-ethylcarbodiimide hydrochloride (ECDI·HCl) and 1-hydroxybenzotriazole (HOBt) to give new thiazolidine-4-one derivatives **7a**–**l** with pyrazolin-5-one moieties.

The structures of the compounds was assigned on the basis of spectral data (IR, ^1^H-NMR, ^13^C-NMR, MS) which are provided in the Experimental Section. In the IR spectra of ethyl 3-(2-aryl-4-oxo-thiazolidin-3-yl)-propionates **4a**–**l** the appearance of the C=O stretching band of the thiazolidine-4-one rings at 1676–1654 cm^−1^, together with the characteristic C-S absorption band at 648–632 cm^−1^ confirm the success of the cyclization reaction and the formation of the thiazolidine system. For these compounds the characteristic absorption band of the ester group appears in the 1728–1712 cm^−1^ region and this band disappears in the spectra of corresponding acids **5a**–**l** in which the characteristic carboxyl group absorption band was observed in 1743–1662 cm^−1^ region. The characteristic absorption band of the amide bond appears in the spectra of pyrazoline-thiazolidine-4-one derivatives **7a**–**l** in the 1686–1652 cm^−1^ region.

The formation of the thiazolidine-4-one heterocycle system has also been proved by the characteristic NMR data. In the ^1^H-NMR spectra of compounds **4a**–**l** the CH (SCHN) proton resonates between 6.17–5.50 ppm as a singlet, doublet or multiplet, depending of the substitution of the phenyl ring. The protons of the methylene group (-CH_2_-S) appears as two sets of signals. One proton resonates as a multiplet between 3.82–3.47 ppm and the second one resonates as a doublet, doublet of doublets and doublet of triplets between 3.67–3.38 ppm. The carbons of the thiazolidine-4-one system appear in the ^13^C-NMR spectra between 64.17–58.26 ppm and 39.18–32.59 ppm, respectively.

The carboxyl group proton of the thiazolidine-propanoic acid derivatives **5a**–**l** resonates as a singlet between 12.35–10.12 ppm. In the ^1^H-NMR spectra of the pyrazoline-thiazolidine-4-one derivatives **7a**–**l**, the amide bond proton resonates as single, doublet or multiplet between 9.67–8.96 ppm. Moreover the presence of the pyrazoline system was proved by the proton signals of two methyl groups, which resonate as singlets at 3.12–3.08 ppm and 2.33–2.16 ppm, respectively. The carbons of the pyrazoline ring appear in the ^13^C-NMR spectra between 150.65–150.45 ppm and 108.38–107.83 ppm. The proton and carbon signals for other characteristic groups were all observed according to the expected chemical shift and integral values. This NMR spectral data, coupled with the corresponding mass spectra, lend strong support to the proposed structures of the all the synthesized compounds.

### 2.2. Biological Evaluation

#### 2.2.1. Ferric Reducing Antioxidant Power (FRAP) Assay

The ferric reducing antioxidant power assay is a simple and sensitive method used to evaluate the antioxidant potential of compounds. In the presence of the electron-donating compounds, the potassium ferric/ferricyanide complex is reduced to its ferrous form (Fe^2+^) which is complexed with ferric chloride to form a blue colored complex. The amount of this complex is quantitatively determined by measuring the intensity of colour at 700 nm [24]. The reaction between the ferrous form and the ferric chloride is:

4FeCl_3_ + 3K_4_[Fe(CN)_6_] → Fe_4_[Fe(CN)_6_]_3_ + 12KCl (1)

**Figure 1:**
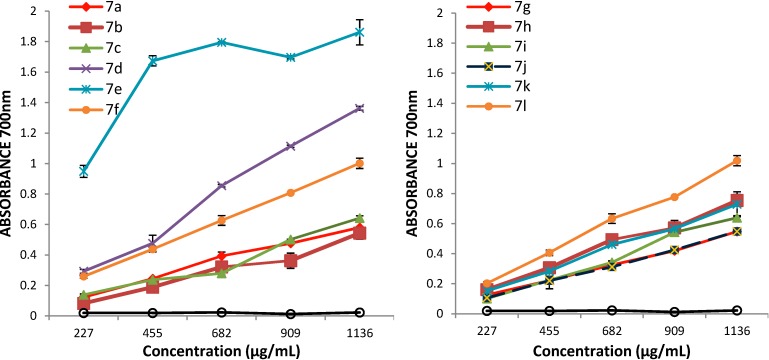
The absorbance of the derivatives **7a**–**l** in reference to phenazone.

The absorbance value of the samples at different concentrations (10 mg/mL, 8 mg/mL, 6 mg/mL, 4 mg/mL, 2 mg/mL in DMSO) are presented in Figure 1. The results expressed as EC_50_ values (mg/mL) are shown in Table 2. Low EC_50_ values indicate a higher ferric reducing antioxidant power. As we expected, the absorbance of the sample increased with the concentration, which means that reducing power of the tested compounds is concentration-dependent. The analysis of the obtained data revealed that the chemical modulation of the pyrazoline-5-one moiety through introduction of thiazolidine-4-one rings via a propioanamide chain has a great influence on antioxidant potential; all tested compounds were more active than phenazone, which was used as reference. Because phenazone showed a very low absorbance at the same concentrations as the tested compounds **7a**–**l**, an EC_50_ could not be determined for it. It was also observed that the activity of the tested compounds depends on the substituents on the thiazolidine-4-one phenyl ring. The most active compound was **7e**, which has a 2-OCH_3_ substituent on the phenyl ring. This compound has EC_50_ = 0.122 ± 0.003, which means that it is about eight time more active than the unsubstituted compound **7a** (EC_50_ = 0.9647 ± 0.0108). Good activity was also shown by compounds **7d** (4-Br, EC_5_ = 0.4653 ± 0.0334), **7f** (3-OCH_3_, EC_50_ = 0.5316 ± 0.0063) and **7l** (4-CH_3_; EC_50_ = 0.5455 ± 0.0177), being about twice as active as **7a**. The compounds **7h** (2-NO_2_), **7i** (3-NO_2_) and **7k** (3-OCH_3_, 4-OH) also have good activity in reference to **7a**. All tested compounds are less active than vitamin E at the same concentration used as positive control.

**Table 2:** The ferric reducing antioxidant power (EC_50_, mg/mL) of the derivatives **7a**–**l**.

Sample	EC_50_, mg/mL	Sample	EC_50_, mg/mL
**7a**	0.9647 ± 0.0108	**7g**	1.1080 ± 0.0256
**7b**	1.0817 ± 0.0413	**7h**	0.6895 ± 0.0132
**7c**	0.9073 ± 0.0021	**7i**	0.8648 ± 0.0322
**7d**	0.4653 ± 0.0334	**7j**	1.0634 ± 0.0441
**7e**	0.1221 ± 0.0025	**7k**	0.8042 ± 0.0130
**7f**	0.5316 ± 0.0063	**7l**	0.5455 ± 0.0177
**Phenazone**	nd	**Vitamin E**	0.0143 ± 0.0027

Data are mean ± SD (n = 3, *p* < 0.05).

#### 2.2.2. Phosphomolydenum Reducing Antioxidant Power (PRAP) Assay

**Figure 2:**
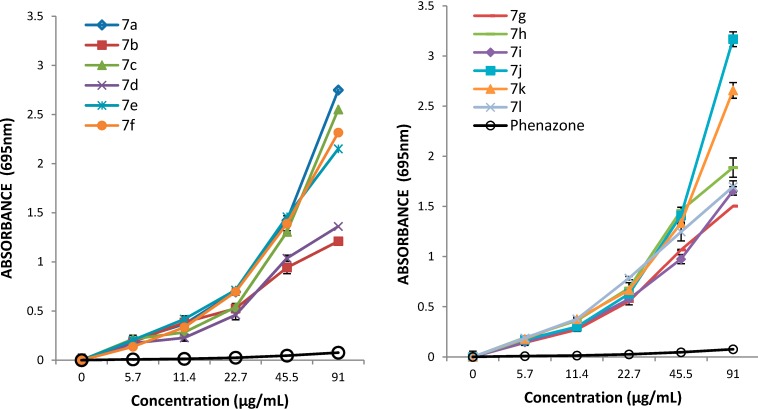
The absorbance of the derivatives **7a**–**l** in reference with phenazone.

This assay is based on quantitative monitoring of phophomolybdenum blue complex which presents a maximum absorption band at 695 nm [25]. The absorbance value of the samples at different concentrations (1 mg/mL, 0.5 mg/mL, 0.25 mg/L, 0.125 mg/mL, 0.0625 mg/mL in DMSO) are presented in Figure 2. The results expressed as EC_50_ values (mg/mL) are shown in Table 3. Low values of EC_50_ demonstrate a higer phosphomolydenum reducing antioxidant power.

The data of this assay also support the conclusion that the antioxidant activity of the tested compound increases with concentration and that all tested compounds are more active than phenazone. It was observed that the presence of a 2-OCH_3_ substituent on the thiazolidine-4-one phenyl ring also has a good influence on antioxidant properties, the corresponding compound **7e** being the most active (EC_50_ = 0.0138 ± 0.0029). In comparison with the unsubstituted compound **7a** (EC_50_ = 0.0153 ± 0.0010) this compound was slightly more active. The 4-CH_3_ and 2-NO_2_ substituents also had a good influence on the reducing antioxidant power, as the corresponding compounds **7l** (EC_50_ = 00.0143 ± 0.0038) and **7h** (0.0146 ± 0.0016) were also slightly more active than **7a**. In this assay all the tested compounds were more active than vitamin E (0.0304 ± 0.0024) at the same concentrations used as positive control.

**Table 3:** The phosphomolydenum reducing antioxidant power (EC_50_ mg/mL) of **7a**–**l**.

Sample	EC_50_ mg/mL	Sample	EC_50_ mg/mL
**7a**	0.0153 ± 0.0010	**7g**	0.0222 ± 0.0043
**7b**	0.0223 ± 0.0019	**7h**	0.0146 ± 0.0016
**7c**	0.0209 ± 0.0020	**7i**	0.0220 ± 0.0016
**7d**	0.0248 ± 0.0020	**7j**	0.0182 ± 0.0080
**7e**	0.0138 ± 0.0029	**7k**	0.0163 ± 0.0025
**7f**	0.0166 ± 0.0017	**7l**	0.0143 ± 0.0038
**Phenazone**	nd	**Vitamin E**	0.0304 ± 0.0024

Data are mean ± SD (n = 3, *p* < 0.05).

#### 2.2.3. DPPH Radical Scavenging Assay

**Figure 3:**
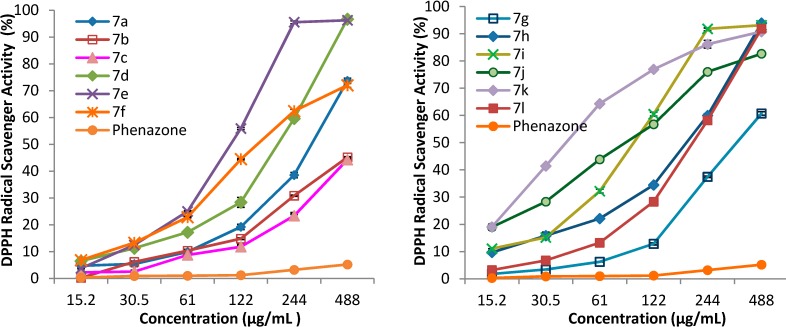
The DPPH radical scavenging ability (%) of the derivatives **7a**–**l**.

DPPH (1,1-diphenyl-2-picrylhydrazyl) is a well-known radical which reacts with different antioxidant compounds whereby its deep violet color in methanol solution changes to yellow. The antioxidant effect is monitored by the decreasing intensity of the absorption band centered at about 515 nm [26]. The DPPH radical scavenging ability (%) of samples at different concentrations (20 mg/mL, 10 mg/mL, 5 mg/mL, 2.5 mg/mL, 1.25 mg/mL, 0.625 mg/mL in DMSO) is presented in Figure 3. Higher scavenging ability values indicate a higher radical scavenging effectiveness. The results expressed as EC_50_ values (mg/mL) are shown in Table 4. Low values of EC_50_ demonstrate a higher scavenging ability.

**Table 4:** The DPPH scavenging ability (EC_50_ mg/mL) of the derivatives **7a**–**l**.

Sample	EC_50_ mg/mL	Sample	EC_50_ mg/mL
**7a**	0.3050 ± 0.0026	**7g**	0.3542 ± 0.0049
**7b**	0.6161 ± 0.0069	**7h**	0.1858 ± 0.0031
**7c**	0.5892 ± 0.0099	**7i**	0.0943 ± 0.0016
**7d**	0.2056 ± 0.0029	**7j**	0.0849 ± 0.0043
**7e**	0.1685 ± 0.0005	**7k**	0.0390 ± 0.0006
**7f**	0.1513 ± 0.0020	**7l**	0.2017 ± 0.0025
**Phenazone**	nd	**Vitamin E**	0.0011 ± 0.0002

Data are mean ± SD (n = 3, *p* < 0.05).

The chemical modulation of the pyrazoline-5-one ring through introduction of thiazolidine-4-one rings via a propioanamide chain improves the DPPH radical scavenging ability, as all tested compounds were more active than phenazone. For phenazone at 20 mg/mL (488 µg/mL in the tube test) DPPH scavenging ability was only 5.19% ± 0.40%. The scavenging ability depends on the phenyl ring substituent of the thiazolidine-4-one. Among the tested compounds the most active was **7k** (3-OCH_3_, 4-OH,) with EC_50_ = 0.0390 ± 0.0006, this compound being about eight time more active than the unsubstituted compound **7a** (EC_50_ = 0.3050 ± 0.0026). A high influence was also shown by the substituents 3-OH/4-OCH_3_ and 3-NO_2_, as the corresponding compounds **7j** (EC_50_ = 0.0849 ± 0.0043) and **7i** (EC_50_ = 0.0943 ± 0.0016) were 3.6 and 3.2 times more active than **7a** respectively. Good scavenging ability was also shown by **7f** (EC_50_ = 0.1513 ± 0.0020), **7e** (EC_50_ = 0.1685 ± 0.0005), **7h** (EC_50_ = 0.1858 ± 0.0031), **7l** (EC_50_ = 0.2017 ± 0.0025) and **7d** (EC_50_ = 0.2056 ± 0.0029), the compounds being about twice (**7f**, **7e**, **7h**) and 1.5 times (**7l**, **7d**) more active than **7a**, respectively. All tested compounds were less active than the vitamin E at the same concentration used as positive control.

#### 2.2.4. The ABTS Radical Scavenging Assay

The ABTS (2,2'-azino-bis(3-ethylbenzothiazoline-6-sulfonic acid) radical scavenging assay is a rapid and efficient method, based on the ability of the hydrogen donating antioxidants to scavenge the long-life radical cation ABTS^+^. The ABTS^+^ is generated by the reaction between 2,2'-azino-bis(3-ethylbenzothiazoline-6-sulfonic acid) and ammonium persulfate. The scavenging ability of the compounds is monitored by the decrease of the intensity of the blue colour of the ABTS^+^ species which presents a maximum absorption band centered at about 734 nm [27].

**Figure 4:**
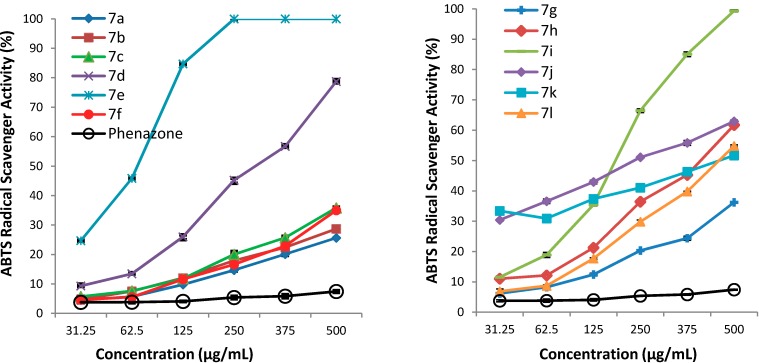
The ABTS radical scavenging ability (%) of the derivatives **7a**–**l**.

The ABTS radical scavenging ability (%) of samples at different concentrations (20 mg/mL, 10 mg/mL, 5 mg/mL, 2.5 mg/mL, 1.25 mg/mL, 0.625 mg/mL in DMSO) is presented in Figure 4. Higher scavenging ability values indicate a higher potential radical scavenging effectiveness. The results expressed as EC_50_ values (mg/mL) are shown in Table 5. Low EC_50_ values indicate a higher scavenging ability.

**Table 5:** The ABTS scavenging ability (EC_50_ mg/mL) of the derivatives **7a**–**l**.

Sample	EC_50_ mg/mL	Sample	EC_50_ mg/mL
**7a**	0.9340 ± 0.0251	**7g**	0.6677 ± 0.0160
**7b**	0.8874 ± 0.0322	**7h**	0.4074 ± 0.0012
**7c**	0.7020 ± 0.0372	**7i**	0.1729 ± 0.0020
**7d**	0.2960 ± 0.0067	**7j**	0.2190 ± 0.0097
**7e**	0.0671 ± 0.0010	**7k**	0.4570 ± 0.0113
**7f**	0.6800 ± 0.0191	**7l**	0.4556 ± 0.0050
**Phenazone**	nd	**Vitamin E**	0.0072 ± 0.0002

Data are mean ± SD (n = 3, *p* < 0.05).

From the data presented in Figure 4 it is obvious that the ABTS radical scavenging ability of all tested compounds **7a**–**l** are higher than that of phenazone, which at 20 mg/mL (500 µg/mL in the tube test) showed a scavenging ability of 7.45 ± 0.33%. Moreover, the phenyl ring substitution of the thiazolidine-4-one improves the scavenging ability, as all substituted compounds **7b**–**l** were more active than **7a**. The most active compound was **7e** (2-OCH_3_, EC_50_ = 0.0671 ± 0.0010) which was 14 times more active than **7a** (EC_50_ = 0.9340 ± 0.0251). Very good scavenging ability was also shown by **7i** (3-NO_2_, EC_50_ = 0.1729 ± 0.0020), **7j** (3-OH, 4-OCH_3_, 0.2190 ± 0.0097) and **7d** (4-Br, 0.2960 ± 0.0067); these compounds were about 5.5, 4 and 3 times more active, respectively, than **7a**. At the same concentration all tested compounds are less active than vitamin E used as positive control.

## 3. Experimental Section

### 3.1. General Experimental Procedures

The melting points were measured using a Buchi Melting Point B-540 apparatus (Büchi Labortechnik AG, Postfach, Switzerland) and they are uncorrected. The FT-IR spectra were recorded on Horizon MB^TM^ FT-IR (ABB Analytical Measurement, Québec, Canada), over a 500–4000 cm^−1^ range, after 32 scans at a resolution of 4 cm^−1^. The spectra processing was carried out with the Horizon MB^TM^ FTIR Software. The ^1^H-NMR and ^13^C-NMR spectra were obtained on a Bruker Avance400 MHz Spectrometer (Brucker, Wissemboug, France) using tetramethylsilane as internal standard and CDCl_3_ as solvent, unless otherwise specified. The chemical shifts are shown in δ values (ppm). The mass spectra were registered using a Bruker MaXis Ultra-High Resolution Quadrupole Time-of-Flight Mass Spectrometer (Brucker Daltonik GmbH, Bremen, Germany). The progress of reaction was monitored on TLC, using pre-coated Kieselgel 60 F254 plates (Merck KGaA, Darmstadt, Germany) and the compounds were visualized using UV light.

### 3.2. Synthetic Procedures

#### 3.2.1. Preparation of Ethyl 3-(2-Aryl-4-oxo-thiazolidin-3-yl)-propionates **4a**–**l**

To a solution of ethyl 3-aminopropionate hydrochloride **2** (10 mmol) in freshly distilled toluene (15 mL), aromatic aldehydes (15 mmol) were added under an inert atmosphere according to the procedure described for other compounds [28]. The mixture was stirred for 5 min and mercaptoacetic acid **3** (20 mmol) was added. After 5 min, *N,N*-diisopropylethylamine (DIPEA, 13 mmol) was added and then the mixture was heated at 110–115 °C for 36 h until completion of the reaction (TLC monitoring, using ethyl acetate/petroleum ether, 4:6, v/v, UV light at 254 nm). The mixture was neutralized with saturated solution of sodium bicarbonate and extracted with ethyl acetate (2 × 25 mL). The organic layer was separated and washed with hydrochloric acid 1M and then with saturated solution of sodium chloride. Finally, the organic layer was dried over MgSO_4_ and filtered. The solvent was removed under reduced pressure and the residue was purified on a silica gel column using ethyl acetate/petroleum ether (4:6, v/v) as eluent system.

*Ethyl 3-(2-phenyl-4-oxothiazolidin-3-yl)propanoate* (**4a**). Yield: 56%, yellow liquid; IR (ATR diamond, cm^−1^): 2980 (CH_Ar_), 1728 (CO_ester_), 1674 (CO_thiazolidine-4-one_), 639 (C-S); ^1^H-NMR: 7.38–7.26 (m, 5H, Ar-H), 5.71 (s, 1H, CH), 4.10–4.02 (m, 2H, CH_2_CH_3_), 3.79–3.69 (m; 1H, CH_2_S; 1H, CH_2_N) 3.63 (dt, *J* = 15.6, 1.7Hz, 1H, CH_2_S), 3.05 (dtd, *J* = 14.1, 7.1, 1.7 Hz, 1H, CH_2_N), 2.66–2.56 (m, 1H, CH_2_CO), 2.40–2.31 (m, 1H, CH_2_CO), 1.2 (tt, *J* = 7.1, 1.7 Hz, 3H, CH_3_); ^13^C-NMR: 171.40, 171.35 (2C, CO), 139.54 (C_Ar_), 129.19 (2C, CH_Ar)_, 129.09 (2C, CH_Ar_), 127.00 (CH_Ar_), 63.82 (CH), 60.75 (CH_2_CH_3_), 39.08 (CH_2_S), 32.63 (CH_2_N), 31.92 (CH_2_CO), 14.12 (CH_3_); HRMS (EI-MS): *m/z* Calcd for C_14_H_17_NO_3_S: 280.1002 [M+H]^+^, Found: 280.1004 [M+H]^+^.

*Ethyl 3-[2-(4-chlorophenyl)-4-oxothiazolidin-3-yl]propanoate* (**4b**). Yield: 46%, colorless liquid, IR (ATR diamond, cm^−1^): 2980 (CH_Ar_), 1727 (CO_ester_), 1674 (CO_thiazolidine-4-one_), 767 (C-Cl), 622 (C-S) [29]; ^1^H-NMR: 7.22–7.09 (m, 4H, Ar-H), 5.59 (d, *J* = 2.0 Hz, 1H, CH), 3.92 (q, *J* = 7.1 Hz, 2H, CH_2_CH_3_), 3.67–3.52 (m; 1H, CH_2_S, 1H, CH_2_N), 3.48 (d, *J* = 15.5 Hz, 1H, CH_2_S), 2.88 (dt, *J* = 14.2, 7.2 Hz, 1H, CH_2_N), 2.48 (dt, *J* = 16.6, 7.2 Hz, 1H, CH_2_CO), 2.23(dt, *J* = 16.6, 6.3 Hz, 1H, CH_2_CO), 1.05 (t, *J* = 7.2 Hz, 3H, CH_3_); ^13^C-NMR: 171.15, 171.01 (2C, CO), 138.28, 134.66 (2C, C_Ar_), 129.12 (2C, CH_Ar_), 128.46 (CH_Ar_), 62.88 (CH), 60.61 (CH_2_CH_3_), 38.91 (CH_2_S), 32.37 (CH_2_N), 31.80 (CH_2_CO), 14.03 (CH_3_); HRMS (EI-MS): *m/z* Calcd for C_14_H_16_ClNO_3_S: 314.0612 [M+H]^+^, Found: 314.0614 [M+H]^+^.

*Ethyl 3-[2-(4-fluorophenyl)-4-oxothiazolidin-3-yl]propanoate* (**4c**). Yield: 42%, colorless liquid, IR (ATR diamond, cm^−1^): 2981 (CH_Ar_), 1727 (CO_ester_), 1674 (CO_thiazolidine-4-one_), 1155 (C-F), 636 (C-S) [29]; ^1^H-NMR: 7.33–7.25 (m, 2H, Ar-H), 7.03 (t, *J* = 8.6 Hz, 2H, Ar-H), 5.71 (d, *J* = 2.0 Hz, 1H, CH), 4.06 (qd, *J* = 7.1, 0.9 Hz, 2H, CH_2_CH_3_ ), 3.77–3.59 (m; 2H, CH_2_S; 1H, CH_2_N), 3.01 (dt, *J* = 14.3, 7.1 Hz, 1H, CH_2_N), 2.60 (dt, *J* = 16.4, 7.1 Hz, 1H, CH_2_CO), 2.34 (dt, *J* = 16.4, 6.3 Hz, 1H, CH_2_CO), 1.22–1.13 (m, 3H, CH_3_); ^13^C-NMR: 171.32, 171.17 (2C, CO), 164.14/161.67, 135.31/135.28 (2C, C_Ar_), 129.05, 128.96 (2C, CH_Ar_), 116.11, 115.90 (2C, CH_Ar_), 63.10 (CH), 60.72 (CH_2_CH_3_), 38.90 (CH_2_S), 32.50 (CH_2_N), 31.84 (CH_2_CO), 14.03 (CH_3_); HRMS (EI-MS): *m/z* Calcd for C_14_H_16_FNO_3_S: 298.0900 [M+H]^+^, Found: 298.0909 [M+H]^+^.

*Ethyl 3-[2-(4-bromophenyl)-4-oxothiazolidin-3-yl]propanoate* (**4d**). Yield: 76%, light yellow liquid; IR (ATR diamond, cm^−1^): 2979 (CH_Ar_), 1727 (CO_ester_), 1674 (CO_thiazolidine-4-one_), 625 (C-S) [29]; ^1^H-NMR: 7.39 (dd, *J* = 8.5, 1.9 Hz, 2H, Ar-H), 7.11 (dd, *J* = 8.5, 1.9 Hz, 2H, Ar-H), 5.62 (d, *J* = 1.9 Hz, 1H, CH), 4.01–3.94 (m, 2H, CH_2_CH_3_), 3.68–3.58 (m;1H, CH_2_S; 1H, CH_2_N), 3.57–3.50 (m, 1H, CH_2_S), 2.93 (m, 1H, CH_2_N), 2.58–2.49 (m, 1H, CH_2_CO), 2.34–2.23 (m, 1H, CH_2_CO), 1.11 (td, *J* = 7.1, 1.9 Hz, 3H, CH_3_); ^13^C-NMR: 171.38, 171.27 (2C, CO), 138.90, 123.14 (2C, C_Ar_), 132.29 (2C, CH_Ar_), 128.88 (2C, CH_Ar_), 63.20 (CH), 60.85 (CH_2_CH_3_), 39.11 (CH_2_N), 32.59 (CH_2_S), 32.01 (CH_2_CO), 14.23 (CH_3_); HRMS (EI-MS): *m/z* Calcd for C_14_H_16_BrNO_3_S: 358.0107 [M+H]^+^, Found: 358.0106 [M+H]^+^.

*Ethyl 3-[2-(2-methoxyphenyl)-4-oxothiazolidin-3-yl]propanoate* (**4e**). Yield: 82%, yellow liquid; IR (ATR diamond, cm^−1^): 2979 (CH_Ar_), 1727 (CO_ester_), 1674 (CO_thiazolidine-4-one_), 644 (C-S) [29]; ^1^H-NMR (400 MHz, CDCl_3_, δ ppm): 7.19 (ddd, *J* = 8.3, 7.5, 1.7 Hz, 1H, Ar-H), 7.01 (dd, *J* = 7.5, 1.7 Hz, 2H, Ar-H), 6.87–6.79 (m, 1H, Ar-H), 5.94 (d, *J* = 2.0 Hz, 1H, CH), 4.02–3.95 (m, 2H, CH_2_CH_3_), 3.80–3.71 (m; 3H, OCH_3_; 1H, CH_2_N), 3.59 (dd, *J* = 15.4, 2.0 Hz, 1H, CH_2_S), 3.44 (d, *J* = 15.4 Hz, 1H, CH_2_S), 2.97 (dt, *J* = 14.2, 7.4 Hz, 1H, CH_2_N), 2.54 (dt, *J* = 16.5, 7.4 Hz, 1H, CH_2_CO), 2.34 (ddd, *J* = 16.4, 7.4, 6.0 Hz, 1H, CH_2_CO), 1.11 (t, *J* = 7.4 Hz, CH_3_); ^13^C-NMR: 171.72, 170.93 (2C, CO), 156.63, 127.37 (2C, C_Ar_), 129.64, 126.33, 120.53, 110.85 (4C, CH_Ar_), 60.35 (CH_2_CH,_3_), 58.26 (CH), 55.30 (CH_3_O), 39.00 (CH_2_S), 32.11 (CH_2_N), 31.77 (CH_2_CO), 13.84 (CH_3_); HRMS (EI-MS): *m/z* Calcd for C_15_H_19_NO_4_S: 310.1107 [M+H]^+^, Found: 310.1111 [M+H]^+^.

*Ethyl 3-[2-(3-methoxyphenyl)-4-oxothiazolidin-3-yl]propanoate* (**4f**). Yield: 72%, slightly yellow liquid; IR (ATR diamond, cm^−1^): 2979 (CH_Ar_), 1727 (CO_ester_), 1674 (CO_thiazolidine-4-one_), 645 (C-S); ^1^H-NMR: 7.29 (t, *J* = 7.9 Hz, 1H, Ar-H), 6.88 (ddd, *J* = 7.9, 3.0, 1.6 Hz, 2H, Ar-H), 6.84 (t, *J* = 2.0 Hz, 1H, Ar-H), 5.71 (d, *J* = 2.0 Hz, 1H, CH), 4.10 (qd, *J* = 7.2, 1.0 Hz, 2H, CH_2_CH_3_), 3.82–3.75 (m; 3H, OCH_3_; 1H, CH_2_-N; 1H, CH_2_S), 3.67 (d, *J* = 15.6 Hz, 1H, CH_2_S), 3.11 (dt, *J* = 14.2, 7.2 Hz, 1H, CH_2_N), 2.64 (dt, *J* = 16.2, 7.2 Hz, 1H, CH_2_CO), 2.41 (dt, *J* = 16.2, 6.4 Hz, 1H, CH_2_CO), 1.23 (t, *J* = 7.2 Hz, 3H, CH_3_); ^13^C-NMR: 171.55, 171.38 (2C, CO), 160.16, 141.12 (2C, C_Ar_), 130.21, 119.13, 114.61, 112.43 (4C, CH_Ar_), 63.82 (CH), 60.80 (CH_2_CH_3_), 55.33 (CH_3_O), 39.18 (CH_2_S), 32.64 (CH_2_N), 31.95 (CH_2_CO), 14.13 (CH_3_); HRMS (EI-MS): *m/z* Calcd for C_15_H_19_NO_4_S: 310.1108 [M+H]^+^, Found: 310.1110 [M+H]^+^.

*Ethyl 3-[2-(4-methoxyphenyl)-4-oxothiazolidin-3-yl]propanoate* (**4g**). Yield: 71%, light yellow liquid; IR (ATR diamond, cm^−1^): 2979 (CH_Ar_), 1728 (CO_ester_), 1673 (CO_thiazolidine-4-one_), 628 (C-S) [29]; ^1^H-NMR: 7.09–7.05 (m, 2H, Ar-H), 6.70–6.66 (m, 2H, Ar-H), 5.50 (d, *J* = 1.9 Hz, 1H, CH), 3.89–3.83 (m, 2H, CH_2_CH_3_), 3.57–3.55 (m, 3H, OCH_3_), 3.53–3.47 (m; 1H, CH_2_S; 1H, CH_2_N), 3.42 (dd, *J* = 15.6, 2.8 Hz, 1H, CH_2_S), 2.91–2.82 (m, 1H, CH_2_N), 2.40 (m, 1H, CH_2_CO), 2.19–2.10 (m, 1H, CH_2_CO), 1.02–0.97 (m, 3H, CH_3_); ^13^C-NMR: 171.67, 170.99 (2C, CO), 160.06, 130.67 (2C, C_Ar_), 128.10 (2C, CH_Ar_), 113.81 (2C, CH_Ar_), 62.86 (CH), 60.04 (CH_2_CH_3_), 54.70 (CH_3_O), 38.35 (CH_2_S), 32.09 (CH_2_N), 31.29 (CH_2_CO), 13.57 (CH_3_); HRMS (EI-MS): *m/z* Calcd for C_15_H_19_NO_4_S: 310.1108 [M+H]^+^, Found: 310.1110 [M+H]^+^.

*Ethyl 3-[2-(2-nitrophenyl)-4-oxothiazolidin-3-yl]propanoate* (**4h**). Yield: 95%, yellow liquid; IR (ATR diamond, cm^−1^): 2981 (CH_Ar_), 1726 (CO_ester_), 1676 (CO_thiazolidine-4-one_), 641 (C-S), 1524 (sim NO_2_), 1343 (asim NO_2_); ^1^H-NMR: 7.92 (dt, *J* = 8.0, 2.4 Hz, 1H, Ar-H), 7.61–7.54 (m, 1H, Ar-H), 7.40–7.33 (m, 1H, Ar-H), 7.19 (dq, *J* = 8.0, 1.5 Hz, 1H, Ar-H), 6.17 (t, *J* = 1.9 Hz, 1H, CH), 3.88 (dd, *J* = 7.2, 3.8 Hz, 2H, CH_2_CH_3_), 3.83–3.73 (m, 1H, CH_2_N), 3.55–3.47 (m, 1H, CH_2_S), 3.38 (dd, *J* = 15.8, 3.8 Hz, 1H, CH_2_S), 2.93–2.83 (m, 1H, CH_2_N), 2.56–2.45 (m, 1H, CH_2_CO), 2.43–2.32 (m, 1H, CH_2_CO), 1.05–0.98 (m, 3H, CH_3_); ^13^C-NMR: 171.81, 170.79 (2C, CO), 146.62, 136.28 (2C, C_Ar_), 134.18, 128.81, 125.65, 125.26 (4C, CH_Ar_), 60.31 (CH_2_CH_3_), 58.42 (CH), 38.99 (CH_2_S), 31.59 (CH_2_CO), 30.82 (CH_2_N), 13.58 (CH_3_); HRMS (EI-MS): *m/z* Calcd for C_14_H_16_N_2_O_5_S: 325.0853 [M+H]^+^, Found: 325.0854 [M+H]^+^.

*Ethyl 3-[2-(3-nitrophenyl)-4-oxothiazolidin-3-yl]propanoate* (**4i**). Yield: 36%, white solid, m.p. 84–85 °C; IR (ATR diamond, cm^−1^): 2982 (CH_Ar_), 1727 (CO_ester_), 1676 (CO_thiazolidine-4-one_), 1528 (sym. NO_2_), 1349 (asym. NO_2_), 639 (C-S); ^1^H-NMR: 8.15–8.04 (m, 2H, Ar-H), 7.63 (dt, *J* = 8.0, 1.4 Hz, 1H, Ar-H), 7.52 (t, *J* = 7.8 Hz, 1H, Ar-H), 5.83 (d , *J* = 2.0 Hz, 1H, CH), 3.99 (d, *J* = 7.2 Hz, 2H, CH_2_CH_3_), 3.79–3.66 (m; 1H, CH_2_S; 1H, CH_2_N), 3.59 (d, *J* = 15.6 Hz,1H, CH_2_S), 3.01–2.89 (m, 1H, CH_2_N), 2.64–2.53 (m, 1H, CH_2_CO), 2.35 (m, 1H, CH_2_CO), 1.11 (t, *J* = 7.2 Hz, 3H, CH_3_); ^13^C-NMR: 171.12, 170.98 (2C, CO), 148.31, 142.16 (2C, C_Ar_), 132.85, 130.05, 123.73, 121.82 (4C, CH_Ar_), 62.43 (CH), 60.59 (CH_2_CH_3_), 38.85 (CH_2_S), 32.14 (CH_2_N), 31.64 (CH_2_CO), 13.83 (CH_3_); HRMS (EI-MS): *m/z* Calcd for C_14_H_16_N_2_O_5_S: 325.0852 [M+H]^+^, Found: 325.0853 [M+H]^+^.

*Ethyl 3-{2-[(3-hydroxy-4-methoxy)phenyl]-4-oxothiazolidin-3-yl}propanoate* (**4j**). Yield: 56%, white solid, m.p. 74–75 °C; IR (ATR diamond, cm^−1^): 3422 (OH), 2930 (CH_Ar_), 1712 (CO_ester_), 1654 (CO_thiazolidine-4-one_), 648 (C-S); ^1^H-NMR: 6.83 (d, *J* = 1.5 Hz, 1H, Ar-H), 6.76–6.72 (m, 2H, Ar-H; s, 1H, OH), 5.58 (d, *J* = 1.5 Hz, 1H, CH), 4.05–3.99 (m, 2H, CH_2_CH_3_), 3.79 (s, 3H, OCH_3_), 3.73–3.63 (m; 1H CH_2_S; 1H, CH_2_N), 3.58 (d, *J* = 15.6 Hz, 1H, CH_2_S), 3.04 (dt, *J* = 14.2, 7.2 Hz, 1H, CH_2_N), 2.58–2.48 (m, 1H, CH_2_CO), 2.32 (dt, *J* = 16.3, 6.5 Hz, 1H, CH_2_CO), 1.15 (t, *J* = 7.2 Hz, 3H, CH_3_); ^13^C-NMR: 171.35, 171.26 (2C, CO), 147.59, 146.33, 131.85 (3C, C_Ar_), 118.77, 113.17, 110.79 (3C, CH_Ar_), 63.59 (CH), 60.61 (CH_2_CH_3_), 55.82 (OCH_3_), 38.83 (CH_2_S), 32.49 (CH_2_N), 31.67 (CH_2_CO), 13.90 (CH_3_); HRMS (EI-MS): *m/z* Calcd for C_15_H_19_NO_5_S: 326.10577 [M+H]^+^, Found: 326.1059 [M+H]^+^.

*Ethyl 3-{2-[(3-methoxy-4-hydroxy)phenyl]-4-oxothiazolidin-3-yl}propanoate* (**4k**). Yield: 34%, white solid, m.p. 134–135 °C; IR (ATR diamond, cm^−1^): 3222 (OH), 2999 (CH_Ar_), 1719 (CO_ester_), 1657 (CO_thiazolidine-4-one_), 643 (C-S); ^1^H-NMR: 6.92–6.83 (m, 3H, Ar-H), 6.00 (s, 1H, OH), 5.71–5.68 (m, 1H, CH), 4.15–4.08 (m, 2H, CH_2_CH_3_), 3.90 (s, 3H, OCH_3_), 3.81–3.67 (m; 2H, CH_2_S; 1H, CH_2_N), 3.12 (dt, *J* = 14.2, 7.1 Hz, 1H, CH_2_N), 2.63 (dt, *J* = 16.3, 7.2 Hz, 1H, CH_2_CO), 2.39 (ddd, *J* = 16.3, 6.9, 6.0 Hz, 1H, CH_2_CO), 1.25 (t, *J* = 7.1 Hz, 3H, CH_3_); ^13^C-NMR: 171.40, 171.36 (2C, CO), 147.27, 146.69, 130.64 (3C, C_Ar_), 120.79, 114.53, 109.22 (3C, CH_Ar_), 64.17 (CH), 60.82 (CH_2_CH_3_), 56.06 (OCH_3_), 39.00 (CH_2_S), 32.85 (CH_2_N), 31.91 (CH_2_CO), 14.13 (CH_3_); HRMS (EI-MS): *m/z* Calcd for C_15_H_19_NO_5_S: 326.1057 [M+H]^+^, Found: 326.1059 [M+H]^+^.

*Ethyl 3-[2-(4-methylphenyl)-4-oxothiazolidin-3-yl]propanoate* (**4l**). Yield: 77%, colorless liquid; IR (ATR diamond, cm^−1^): 2980 (CH_Ar_), 1728 (CO_ester_), 1674 (CO_thiazolidine-4-one_), 632 (S-C); ^1^H-NMR: 7.19 (q, *J* = 8.2 Hz, 4H, Ar-H), 5.71 (d, *J* = 2.0 Hz, 1H, CH), 4.09 (qd, *J* =7.1, 1.3 Hz, 2H, CH_2_CH_3_), 3.80–3.71 (m; 1H, CH_2_S; 1H, CH_2_N), 3.64 (d, *J* = 15.5 Hz, 1H, CH_2_S), 3.08 (dt, *J* = 14.2, 7.1 Hz, 1H, CH_2_N), 2.62 (dt, *J* = 16.3, 7.2 Hz, 1H, CH_2_CO), 2.41–2.36 (m, 1H, CH_2_CO), 2.34 (s, 3H, CH_3_), 1.22 (t, *J* = 7.2 Hz, 3H, CH_3_); ^13^C-NMR: 171.12, 171.10 (2C, CO), 138.94, 136.27 (2C, C_Ar_), 129.55 (2C, CH_Ar_), 126.84 (2C, CH_Ar_), 63.47 (CH), 60.51 (CH_2_CH_3_), 38.82 (CH_2_S), 32.47 (CH_2_N), 31.72 (CH_2_CO), 21.00, 13.94 (2C, CH_3_); HRMS (EI-MS): *m/z* Calcd for C_15_H_19_NO_3_S: 294.1158 [M+H]^+^, Found: 294.1161 [M+H]^+^.

#### 3.2.2. Preparation of 3-(2-Aryl-4-oxothiazolidin-3-yl)-propanoic Acids **5a**–**l**

To a solution of ethyl 3-(2-aryl-4-oxothiazolidin-3-yl)-propanate **4a**–**l** (13.2 mmol) in a mixture of EtOH and THF (1:1, 25 mL:25 mL), potassium hydroxide 1 M (26 mmol) was added according to the procedure for alkaline hydrolysis of esters [30]. The mixture of reaction was stirred for 6–10 h at room temperature until completion of the reaction (TLC monitoring, using ethyl acetate/petroleum ether, 4:6, v/v, UV light at 254 nm). After that, the mixture was neutralized with hydrochloric acid 1 M to pH 2, stirred again for another 20 min and finally extracted with ethyl acetate (2 × 25 mL). The organic layer was dried over MgSO_4_ and filtered. The solvent was removed under reduced pressure and the residue was triturated with ethyl ether.

*3-(2-Phenyl-4-oxothiazolidin-3-yl)propanoic acid* (**5a**). Yield: 56%, white solid, m.p. 124 °C; IR (ATR diamond, cm^−1^): 3063 (OH), 1741 (COOH), 1725 (CO_thiazolidine-4-one_), 699 (C-S); ^1^H-NMR: 11.00–10.88 (m, 1H, COOH), 7.42–7.29 (m, 5H, Ar-H), 5.74 (d, 1H, CH), 3.81(dd, *J* = 15.7, 2.0 Hz, 1H, CH_2_S), 3.78–3.68 (m; 1H, CH_2_S; 1H, CH_2_N), 3.10 (dt, *J* = 14.2, 7.1 Hz, 1H, CH_2_N), 2.69 (dt, *J* = 16.9, 7.2 Hz, 1H, CH_2_CO), 2.43 (dt, *J* = 16.9, 6.4 Hz, 1H, CH_2_CO); ^13^C-NMR: 175.99, 171.10 (2C, CO), 139.15 (C_Ar_), 129.41 (2C, CH_Ar_), 129.20 (2C, CH_Ar_), 127.14 (CH_Ar_), 64.90 (CH), 39.05 (CH_2_S), 32.76 (CH_2_N), 31.63 (CH_2_CO); HRMS (EI-MS): *m/z* Calcd for C_12_H_14_NO_3_S: 252.0691 [M+H]^+^, Found: 252.0691 [M+H]^+^.

*3-[2-(4-Chlorophenyl)-4-oxothiazolidin-3-yl]propanoic acid* (**5b**). Yield: 63%, white solid, m.p. 125–126 °C; IR (ATR diamond, cm^−1^): 3088 (OH), 1742 (COOH), 1724 (CO_thiazolidine-4-one_), 768 (C-Cl), 622 (C-S); ^1^H-NMR: 10.17 (s, 1H, COOH), 7.57–7.11 (m, 4H, Ar-H), 5.75 (s, 1H, CH), 3.88–3.65 (m; 2H, CH_2_S; 1H, CH_2_N), 3.18–3.03 (m, 1H, CH_2_N), 2.75–2.64 (m, 1H, CH_2_CO), 2.52–2.30 (s, 1H, CH_2_CO); ^13^C-NMR: 176.11, 172.08 (2C, CO), 138.87, 135.36 (2C, C_Ar_), 129.54 (2C, CH_Ar_), 128.68 (2C, CH_Ar_), 63.62 (CH), 39.13 (CH_2_S), 32.77 (CH_2_N), 31.76 (CH_2_CO); HRMS (EI-MS): *m/z* Calcd for C_12_H_13_ClNO_3_S: 286.0299 [M+H]^+^, Found: 286.0300 [M+H]^+^.

*3-[2-(4-Fluorophenyl)-4-oxothiazolidin-3-yl]propanoic acid* (**5c**). Yield: 70%, white solid, m.p. 94–95 °C; IR (ATR diamond, cm^−1^): 3076 (OH), 1743 (COOH), 1724 (CO_thiazolidine-4-one_), 1223 (C-F), 623 (C-S); ^1^H-NMR: 10.69 (s, 1H, COOH), 7.34 (dd, *J* = 7.5, 4.0 Hz, 2H, Ar-H), 7.15–7.06 (m, 2H, Ar-H), 5.77 (s, 1H, CH), 3.86–3.69 (m; 2H, CH_2_S; 1H CH_2_N), 3.11 (dd, *J* = 14.4, 7.1 Hz, 1H, CH_2_N), 2.77–2.65 (m, 1H, CH_2_CO), 2.46 (dd, *J* = 14.4, 7.8 Hz, 1H, CH_2_CO); ^13^C-NMR: 176.06, 172.10 (2C, CO), 164.44/161.96, 134.98/134.96 (2C, C_Ar_), 129.35, 129.29 (2C, CH_Ar_), 116.42, 116.40 (2C, CH_Ar_), 63.65 (CH), 39.06 (CH_2_S), 32.81 (CH_2_N), 31.73 (CH_2_CO); HRMS (EI-MS): *m/z* Calcd for C_12_H_13_FNO_3_S: 270.0595 [M+H]^+^, Found: 270.0596 [M+H]^+^.

*3-[2-(4-Bromophenyl)-4-oxothiazolidin-3-yl]propanoic acid* (**5d**). Yield: 75%, white solid, m.p. 116–117 °C; IR (ATR diamond, cm^−1^): 3083 (OH), 1741 (COOH), 1724 (CO_thiazolidine-4-one_), 653 (C-Br), 621 (C-S); ^1^H-NMR: 10.12 (s, 1H, COOH), 7.52 (d, *J* = 8.1 Hz, 2H, Ar-H), 7.24–7.14 (m, 2H, Ar-H), 5.71 (d, *J* = 2.1 Hz, 1H, CH), 3.84–3.65 (m; 2H CH_2_S; 1H, CH_2_N), 3.07 (dt, *J* = 14.1, 7.0 Hz, 1H, CH_2_N), 2.69 (dt, *J* = 17.1, 7.0 Hz, 1H, CH_2_CO), 2.45 (dt, *J* = 16.9, 6.2 Hz, 1H, CH_2_CO); ^13^C-NMR: 176.10, 171.99 (2C, CO), 138.32, 132.40 (2C, C_Ar_), 128.85 (2C, CH_Ar_), 123.42 (2C, CH_Ar_), 63.55 (CH), 39.04 (CH_2_S), 32.67 (CH_2_N), 31.71 (CH_2_CO); HRMS (EI-MS): *m/z* Calcd for C_12_H_13_BrNO_3_S: 329.9794 [M+H]^+^, Found: 329.9795 [M+H]^+^.

*3-[2-(2-Methoxyphenyl)-4-oxothiazolidin-3-yl]propanoic acid* (**5e**). Yield: 79%, brown sticky product; IR (ATR diamond, cm^−1^) 2940 (OH), 1724 (COOH), 1634 (CO_thiazolidine-4-one_), 608 (C-S); ^1^H-NMR: 10.18 (s, 1H, COOH), 7.34–7.28 (m, 1H, Ar-H), 7.16–7.12 (m, 1H, Ar-H), 6.98–6.90 (m, 2H, Ar-H), 6.11–6.04 (m, 1H, CH), 3.85 (s, 3H, OCH_3_), 3.83–3.74 (m; 1H, CH_2_S, 1H, CH_2_N), 3.63 (d, *J* = 15.6 Hz, 1H, CH_2_S), 3.11 (dt, *J* = 14.3, 7.3 Hz, 1H, CH_2_N), 2.69 (dt, *J* = 16.9, 7.3 Hz, 1H, CH_2_CO), 2.55–2.46 (m, 1H, CH_2_CO); ^13^C-NMR: 175.48, 172.87 (2C, CO), 156.97, 127.13 (2C, C_Ar_), 130.15, 126.98, 120.88, 111.17 (4C, CH_Ar_), 59.07 (CH), 55.61 (OCH_3_), 39.20 (CH_2_S), 32.63 (CH_2_N), 31.70 (CH_2_CO); HRMS (EI-MS): *m/z* Calcd for C_13_H_16_NO_4_S: 282.0794 [M+H]^+^, Found: 282.0795 [M+H]^+^.

*3-[2-(3-Methoxyphenyl)-4-oxothiazolidin-3-yl]propanoic acid* (**5f**). Yield: 73%, white solid, m.p. 156 °C; IR (ATR diamond, cm^−1^): 2946 (OH), 1723 (COOH), 1627 (CO_thiazolidine-4-one_), 645 (C-S); ^1^H-NMR (DMSO-*d*_6_): 12.34 (s, 1H, COOH), 7.36–7.30 (m, 1H, Ar-H), 6.93 (dt, *J* = 7.8, 1.6 Hz, 3H, Ar-H), 5.82 (d, *J* = 1.9 Hz, 1H, CH), 3.84 (dd, *J* = 15.6, 1.9 Hz, 1H, CH_2_S), 3.76 (s, 3H, OCH_3_), 3.68–3.59 (m; 1H, CH_2_S, 1H, CH_2_N), 2.85 (ddd, *J* = 14.4, 8.7, 6.2 Hz, 1H, CH_2_N), 2.57–2.50 (m, 1H, CH_2_CO), 2.28 (ddd, *J* = 16.4, 8.7, 5.9 Hz, 1H, CH_2_CO); ^13^C-NMR: 172.45, 170.72 (2C, CO), 159.60, 141.95 (2C, C_Ar_), 130.16, 118.87, 114.19, 112.51 (4C, CH_Ar_), 62.03 (CH), 55.19 (OCH_3_), 38.70 (CH_2_S), 31.78 (CH_2_N), 31.40 (CH_2_CO); HRMS (EI-MS): *m/z* Calcd for C_13_H_16_NO_4_S: 282.0794 [M+H]^+^, Found: 282.0797 [M+H]^+^.

*3-[2-(4-Methoxyphenyl)-4-oxothiazolidin-3-yl]propanoic acid* (**5g**). Yield: 83%; white solid, m.p. 108–110 °C; IR (ATR diamond, cm^−1^): 2929 (OH), 1730 (COOH), 1640 (CO_thiazolidine-4-one_), 624 (C-S); ^1^H-NMR (DMSO-*d*_6_):12.35 (s, 1H, COOH), 7.34 (d, *J* =8.2 Hz, 2H, Ar-H), 6.96 (d, *J* = 8.2 Hz, 2H, Ar-H), 5.81 (s, 1H, CH), 3.79 (d, *J* = 14.4 Hz; 3H, OCH_3_; 1H, CH_2_S), 3.69–3.55 (m; 1H, CH_2_S; 1H, CH_2_N), 2.89–2.77 (m, 1H, CH_2_N), 2.47 (d, *J* = 7.6 Hz, 1H, CH_2_CO), 2.25 (dt, *J* = 14.4, 7.6 Hz, 1H, CH_2_CO); ^13^C-NMR: 172.88, 170.91 (2C, CO), 160.06, 132.15 (2C, C_Ar_), 129.05 (2C, CH_Ar_), 114.69 (2C, CH_Ar_), 62.33 (CH), 55.65 (OCH_3_), 38.91 (CH_2_S), 32.37 (CH_2_N), 31.77 (CH_2_CO); HRMS (EI-MS): *m/z* Calcd for C_13_H_16_NO_4_S: 282.079455 [M+H]^+^, Found: 282.0796 [M+H]^+^.

*3-[2-(2-Nitrophenyl)-4-oxothiazolidin-3-yl]propanoic acid* (**5h**). 50%; white solid, m.p. 268 °C; IR (ATR diamond, cm^−1^): 2913 (OH), 1724 (COOH), 1629 (CO_thiazolidine-4-one_), 1515 (sym. NO_2_), 1344 (asym. NO_2_), 674 (C-S); ^1^H-NMR (DMSO-*d*_6_): 12.34 (s, 1H, COOH), 8.12 (dd, *J* = 8.4, 1.3 Hz, 1H, Ar-H), 7.82 (td, *J* = 7.4, 1.3 Hz, 1H, Ar-H), 7.62 (ddd, *J* = 8.4, 7.4, 1.3 Hz, 1H, Ar-H), 7.37 (dd, *J* = 7.8, 1.3 Hz, 1H, Ar-H), 6.26 (d, *J* = 1.7 Hz, 1H, CH), 3.79–3.67 (m; 1H, CH_2_S; 1H, CH_2_N), 3.59 (*J* = 15.7 Hz, 1H, CH_2_S), 2.86 (dt, *J* = 14.2, 7.4 Hz, 1H, CH_2_N), 2.58 (ddd, *J* = 16.7, 7.8, 6.8 Hz, 1H, CH_2_CO), 2.42 (ddd, *J* = 16.7, 7.8, 5.6 Hz, 1H, CH_2_CO); ^13^C-NMR: 172.83, 171.75 (2C, CO), 146.96, 136.64 (2C, C_Ar_), 134.99, 129.38, 126.21, 125.44 (4C, CH_Ar_), 58.04 (CH), 38.89 (CH_2_S), 31.69 (CH_2_N), 30.58 (CH_2_CO); HRMS (EI-MS): *m/z* Calcd for C_12_H_13_N_2_O_5_S: 295.0539 [M+H]^+^, Found: 297.0540 [M+H]^+^.

*3-[2-(3-Nitrophenyl)-4-oxothiazolidin-3-yl]propanoic acid* (**5i**). Yield: 55%, slight brown solid; m.p. 160–162 °C; IR (ATR diamond, cm^−1^): 2917 (OH), 1725 (COOH), 1625 (CO_thiazolidine-4-one_), 1525 (sym. NO_2_), 1350 (asym. NO_2_), 677 (C-S); ^1^H-NMR (DMSO-*d*_6_): 12.33 (s, 1H, COOH), 8.31–8.13 (m, 2H, Ar-H), 7.87 (dt, *J* = 7.9, 1.4 Hz, 1H, Ar-H), 7.71 (t, *J* = 7.9 Hz, 1H, Ar-H), 6.06 (d, *J* = 1.9 Hz, 1H, CH), 3.92 (dd, *J* = 15.4, 1.9 Hz, 1H, CH_2_S), 3.73–3.61 (m; 1H, CH_2_S; 1H, CH_2_N), 2.85 (ddd, *J* = 14.3, 8.4, 6.3 Hz, 1H, CH_2_N), 2.61–2.49 (m, 1H, CH_2_CO), 2.32 (ddd, *J* = 16.6, 8.4, 6.3 Hz, 1H, CH_2_CO); ^13^C-NMR: 172.85, 171.26 (2C, CO), 148.47, 143.44 (2C, C_Ar_), 133.93, 131.14, 124.13, 122.25 (4C, CH_Ar_), 61.42 (CH), 39.13 (CH_2_S), 32.12 (CH_2_N), 31.87 (CH_2_CO); HRMS (EI-MS): *m/z* Calcd for C_12_H_13_N_2_O_5_S: 295.0540 [M+H]^+^, Found: 297.0541 [M+H]^+^.

*3-{2-[(3-Hydroxy-4-methoxy)phenyl]-4-oxothiazolidin-3-yl}propanoic acid* (**5j**). Yield 75%; white solid, m.p. 172 °C; IR (ATR diamond, cm^−1^): 3208 (OH), 1733 (COOH), 1611 (CO_thiazolidine-4-one_), 617 (C-S); ^1^H-NMR (400 MHz, DMSO-*d*_6_, δ ppm): 12.29 (s, 1H, COOH), 9.16 (s, 1H, OH), 6.93–6.87 (m, 1H, Ar-H), 6.80–6.74 (m, 2H, Ar-H), 5.72 (d, *J* = 1.8 Hz, 1H, CH), 3.80–3.74 (m; 3H, OCH_3_, 1H, CH_2_S), 3.64 (d, *J* = 15.4 Hz, 1H, CH_2_S), 3.60–3.52 (m, 1H, CH_2_N), 2.88–2.79 (m, 1H, CH_2_N), 2.47–2.45 (m, 1H, CH_2_CO), 2.25 (m, 1H, CH_2_CO); ^13^C-NMR: 172.44, 170.44 (2C, CO), 148.21, 146.92, 132.12 (3C, C_Ar_), 118.24, 113.75, 111.98 (3C, CH_Ar_), 62.10 (CH), 55.62 (OCH_3_), 38.51 (CH_2_S), 31.91 (CH_2_N), 31.33 (CH_2_CO); HRMS (EI-MS): *m/z* Calcd for C_13_H_16_NO_5_S: 298.07430 [M+H]^+^, Found: 298.0744 [M+H]^+^.

*3-{2-[(3-Methoxy-4-hydroxy)phenyl]-4-oxothiazolidin-3-yl}propanoic acid* (**5k**). Yield 60%, white solid, m.p. 136–137 °C; IR (ATR diamond, cm^−1^): 2946 (OH), 1726 (COOH), 1626 (CO_thiazolidine-4-one_), 615 (C-S); ^1^H-NMR (DMSO-*d*_6_): 12.30 (s, 1H, COOH), 9.22 (s, 1H, OH), 6.93 (s, 1H, Ar-H), 6.87–6.74 (m, 2H, Ar-H), 5.75 (s, 1H, CH), 3.79 (d, *J* = 9.2 Hz; 3H OCH_3_, 1H, CH_2_S), 3.67–3.52 (m; 1H, CH_2_S, 1H, CH_2_N), 2.93–2.82 (m, 1H, CH_2_N), 2.47 (dd, *J* = 9.2, 5.9 Hz, 1H, CH_2_CO), 2.30–2.17 (m, 1H, CH_2_CO); ^13^C-NMR: 172.90, 170.90 (2C, CO), 148.32, 147.63, 130.76 (3C, C_Ar_), 120.43, 115.88, 111.45 (3C, CH_Ar_), 62.91 (CH), 56.14 (OCH_3_), 38.97 (CH_2_S), 32.42 (CH_2_N), 31.80 (CH_2_CO); HRMS (EI-MS): *m/z* Calcd for C_13_H_16_NO_5_S: 298.0743 [M+H]^+^, Found: 298.0744 [M+H]^+^.

*3-[2-(4-Methylphenyl)-4-oxothiazolidin-3-yl]propanoic acid* (**5l**). Yield 71%, white solid, m.p. 119–121 °C; IR (ATR diamond, cm^−1^): 3307 (OH), 1662 (COOH), 1557 (CO_tiazolidine-4-one_), 632 (S-C); ^1^H-NMR (DMSO-*d*_6_): 12.30 (s, 1H, COOH), 7.23 (d, *J* = 7.9 Hz, 2H, Ar-H), 7.15 (d, *J* = 7.9 Hz, 2H, Ar-H), 5.83 (d, *J* = 2.0 Hz, 1H, CH), 3.78 (dd, *J* = 15.4, 2.0 Hz, 1H, CH_2_S), 3.56 (d, 1H, CH_2_S), 3.51 (ddd, *J* = 14.0, 8.9, 5.4 Hz, 1H, CH_2_N), 2.74 (ddd, *J* = 14.0, 8.9, 6.9 Hz, 1H, CH_2_N), 2.31–2.21 (m; 3H, CH_3_; 1H, CH_2_CO), 2.06–1.97 (m, 1H, CH_2_CO); ^13^C-NMR: 176.84, 170.28 (2C, CO), 137.95, 137.75 (2C, C_Ar_), 129.32 (2C, CH_Ar_), 126.98 (2C, CH_Ar_), 62.07 (CH) 38.87 (CH_2_S), 34.25 (CH_2_CO), 31.97 (CH_2_N), 20.80 (CH_3_); HRMS (EI-MS): *m/z* Calcd for C_13_H_16_NO_3_S: 266.0845 [M+H]^+^, Found: 266.0848 [M+H]^+^.

#### 3.2.3. Preparation of the 3-(2-Aryl-4-oxo-thiazolidin-3-yl)-*N*-(2,3-dimethyl-1-phenyl-5-oxo-pyrazolin-4-yl) Propionamide Derivatives **7a**–**l**

4-Aminophenazone (**6**, 3.3 mmol), *N*-(3-dimethylaminopropyl)-*N′*-ethylcarbodiimide hydrochloride (EDCI·HCl) (3.3 mmol) and 1-hydroxybenzotriazole (HOBt) (3.3 mmol) were added to a cold solution of 3-(2-aryl-4-oxo-thiazolidin-3-yl)-propanoic acid **5a**–**l** (3 mmol) in dichloromethane (10–20 mL) under inert atmosphere according to the procedure for amide bond formation [31]. The mixture was stirred for 24 h at room temperature until completion of the reaction (TLC monitoring, using dichloromethane-methanol, 10:0.5–0.8, v/v, UV light at 254). After that, the mixture was washed successively with hydrochloric acid 1M, sodium bicarbonate solution 10% and saturated solution of sodium chloride. The organic layer was collected, dried using anhydrous magnesium sulphate and concentrated by rotary evaporator. The residue was purified on a silica gel column using dichloromethane-methanol, 10:0.5–0.8, v/v as eluent system, and finally the product was triturated with cold ethyl ether.

*3-(2-Phenyl-4-oxothiazolidin-3-yl)-N-(2,3-dimethyl-1-phenyl-5-oxo-pyrazolin-4-yl) propionamide* (**7a**). Yield: 52%, white solid, m.p. 173–175 °C; IR (ATR diamond, cm^−1^): 3183 (NH), 3031 (CH) 1660 (CONH), 1652 (CO_thiazolidine-4-one_), 1651 (CO_pyrazolin-5-one_), 637 (C-S); ^1^H-NMR: 9.17 (s, 1H, NH), 7.47–7.41 (m, 2H, Ar-H), 7.40–7.36 (m, 2H, Ar-H), 7.34–7.27 (m, 4H, Ar-H), 7.25–7.20 (m, 2H, Ar-H), 5.82 (d, *J* = 1.9 Hz, 1H, CH), 3.88 (dt, *J* = 13.6, 6.5 Hz, 1H, CH_2_N), 3.77 (dd, *J* = 15.4, 1.9 Hz, 1H, CH_2_S), 3.66 (d, *J* = 15.4 Hz, 1H, CH_2_S), 3.08 (s, 3H, CH_3_N), 2.95–2.87 (m,1H, CH_2_N), 2.53 (dt, *J* = 15.4, 6.5 Hz, 1H, CH_2_CO), 2.40 (dt, *J* = 15.4, 6.5 Hz, 1H, CH_2_CO), 2.18 (s, 3H, CH_3_); ^13^C-NMR: 171.50, 170.29, 162.16 (3C, CO), 139.91, 134.50 (2C, C_Ar_), 150.60, 108.28 (2C, C_pyrazoline_), 129.40, (2C, CH_Ar_), 129.04 (2C, CH_Ar_), 127.30 (2C, CH_Ar_), 127.09 (2C, CH_Ar_), 124.73, 129.11 (2C, CH_Ar_), 63.44 (CH), 39.53 (CH_2_N), 35.91, 12.16 (2C, CH_3_) 33.12 (CH_2_CO), 32.87 (CH_2_S); HRMS (EI-MS): *m/z* Calcd for C_23_H_25_N_4_O_3_S: 437.1642 [M+H]^+^, Found: 437.1644 [M+H]^+^.

*3-[2-(4-Chlorophenyl)-4-oxothiazolidin-3-yl]-N-(2,3-dimethyl-1-phenyl-5-oxo-pyrazolin-4-yl) propionamide* (**7b**). Yield: 74%, white solid, m.p. 182–183 °C; IR (ATR diamond, cm^−1^): 3172 (NH), 3027 (CH), 1682 (CONH), 1647 (CO_thiazolidine-4-one_), 1616 (CO_pyrazolin-5-one_), 756 (C-Cl), 639 (C-S); ^1^H-NMR: 9.27–9.17 (m, 1H, NH), 7.48–7.43 (m, 2H, Ar-H), 7.39–7.35 (m, 2H, Ar-H), 7.32–7.25 (m, 3H, Ar-H), 7.17 (d, *J* = 8.5 Hz, 2H, Ar-H), 5.81 (d, *J* = 1.8 Hz, 1H, CH), 3.89–3.81 (m, 1H, CH_2_N), 3.78–3.63 (m, 2H, CH_2_S), 3.09 (s, 3H, CH_3_N), 2.86 (dt, *J* = 14.2, 6.5 Hz, 1H, CH_2_N), 2.53 (dt, *J* = 15.3, 6.5 Hz, 1H, CH_2_CO), 2.40 (dt, *J* = 15.3, 6.5 Hz, 1H, CH_2_CO), 2.18 (s, 3H, CH_3_); ^13^C-NMR: 171.26, 170.21, 162.04 (3C, CO), 138.41, 134.71, 134.33 (3C, C_Ar_), 129.34 (2C, CH_A_), 129.19 (2C, CH_A_), 128.51 (2C, CH_A_), 127.31 (2C, CH_A_), 150.47, 108.03 (2C, C_pyrazoline_), 124.71 (CH_Ar_), 62.62 (CH), 39.33 (CH_2_N), 35.76, 12.02 (2C, CH_3_), 33.01 (CH_2_CO), 32.69 (CH_2_S); HRMS (EI-MS): *m/z* Calcd for C_23_H_24_ClN_4_O_3_S: 471.1252 [M+H]^+^, Found: 471.1251 [M+H]^+^.

*3-[2-(4-Fluorophenyl)-4-oxothiazolidin-3-yl]-N-(2,3-dimethyl-1-phenyl-5-oxo-pyrazolin-4-yl) propionamide* (**7c**). Yield: 57%, light yellow solid; m.p. 86–88 °C; IR (ATR diamond, cm^−1^): 3245 (NH), 3013 (CH), 1652 (CONH), 1648 (CO_thiazolidine-4-one_), 1620 (CO_pyrazolin-5-one_), 1150 (C-F), 623 (C-S); ^1^H-NMR: 9.37 (d, *J* = 46.9 Hz, 1H, NH), 7.47–7.41 (m, 2H, Ar-H), 7.38–7.33 (m, 2H, Ar-H), 7.29 (dd, *J* = 7.2, 1.6 Hz, 1H, Ar-H), 7.25–7.18 (m, 2H, Ar-H), 7.01–6.93 (m, 2H, Ar-H), 5.81 (d, *J* = 1.8 Hz, 1H, CH), 3.85 (dd, *J* = 7.7, 6.6 Hz, 1H, CH_2_N), 3.76–3.62 (m, 2H, CH_2_S), 3.08 (s, 3H, CH_3_N), 2.85 (dd, *J* = 13.6, 6.6 Hz, 1H, CH_2_N), 2.56–2.47 (m, 1H, CH_2_CO), 2.44–2.35 (m, 1H, CH_2_CO), 2.16 (s, 3H, CH_3_); ^13^C-NMR: 171.31/171.28, 170.40/170.38, 162.15 (3C, CO), 164.14/161.67, 135.61/135.58 134.35/134.28 (3C, C_Ar_), 129.40 (CH_Ar_), 129.17, 129.08 (2C, CH_Ar_), 127.41 (2C, CH_Ar_), 124.83 (2C, CH_Ar_), 116.00, 115.90 (2C, CH_Ar_), 150.63, 108.03 (2C, C_pyrazoline_), 62.67 (CH), 39.34 (CH_2_N), 35.77, 12.03 (2C, CH_3_), 32.99 (CH_2_CO), 32.80 (CH_2_S); HRMS (EI-MS): *m/z* Calcd for C_23_H_24_FN_4_O_3_S: 455.1542 [M+H]^+^, Found: 455.1543 [M+H]^+^.

*3-[2-(4-Bromophenyl)-4-oxothiazolidin-3-yl]-N-(2,3-dimethyl-1-phenyl-5-oxo-pyrazolin-4-yl) propionamide* (**7d**). Yield: 92%, yellow solid; m.p. 110–112 °C; IR (ATR diamond, cm^−1^): 3196 (NH), 2984 (CH), 1664 (CONH), 1655 (CO_thiazolidine-4-one_), 1621 (CO_pyrazolin-5-one_), 643 (C-S), 668 (C-Br); ^1^H-NMR: 9.67 (s, 1H, NH), 7.40 (ddd, *J* = 13.8, 11.9, 7.6 Hz, 6H, Ar-H), 7.29 (d, *J* = 5.8 Hz, 1H, Ar-H), 7.09 (d, *J* = 8.3 Hz, 2H, Ar-H), 5.80 (s, 1H, CH), 3.90–3.82 (m, 1H, CH_2_N), 3.68 (dd, *J* = 37.7, 15.5 Hz, 2H, CH_2_S), 3.08 (s, 3H, CH_3_N), 2.83–2.75 (m, 1H, CH_2_N), 2.49 (dt, *J* = 12.7, 6.3 Hz, 1H, CH_2_CO), 2.43–2.35 (m, 1H, CH_2_CO), 2.16 (s, 3H, CH_3_); ^13^C-NMR: 171.16, 170.36, 162.11 (3C, CO), 138.99, 134.27, 132.09 (3C, C_Ar_), 129.33 (2C, CH_Ar_), 128.80 (2C, CH_Ar_), 127.33 (2C, CH_Ar_), 124.75 (2C, CH_Ar_), 122.79 (CH_Ar_), 150.65, 107.96 (2C, C_pyrazoline_), 62.49 (CH), 39.29 (CH_2_N), 35.69, 11.96 (2C, CH_3_), 32.84 (CH_2_CO), 32.68 (CH_2_S); HRMS (EI-MS): *m/z* Calcd for C_23_H_24_BrN_4_O_3_S: 515.0747 [M+H]^+^, Found: 515.0745 [M+H]^+^.

*3-[2-(2-Methoxyphenyl)-4-oxo-thiazolidin-3-yl]-N-(2,3-dimethyl-1-phenyl-5-oxo-pyrazolin-4-yl) propionamide* (**7e**). Yield: 60%, white solid; m.p.162 °C; IR (ATR diamond, cm^−1^): 3243 (NH), 2942 (CH), 1675 (CONH), 1647 (CO_thiazolidine-4-one_), 1620 (CO_pyrazolin-5-one_) 617 (C-S); ^1^H-NMR: 9.01 (d, *J* = 6.1 Hz, 1H, NH), 7.41 (dd, *J* = 20.5, 7.8 Hz, 4H, Ar-H), 7.28 (t, *J* = 7.8 Hz, 2H, Ar-H), 7.08 (d, *J* = 7.3 Hz, 1H, Ar-H), 6.90 (d, *J* = 8.5 Hz, 2H, Ar-H), 6.10 (s, 1H, CH), 3.93 (dd, *J* = 13.9, 6.8 Hz, 1H, CH_2_N), 3.87–3.81 (m, 3H, OCH_3_), 3.72 (d, *J* = 15.3 Hz, 1H, CH_2_S), 3.58 (d, *J* = 15.3 Hz, 1H, CH_2_S), 3.08 (s, 3H, CH_3_N), 3.01 (dd, *J* = 13.9, 6.8 Hz, 1H, CH_2_N), 2.59 (dt, *J* = 14.8, 6.8 Hz, 1H, CH_2_CO), 2.48 (dt, *J* = 14.8, 6.8 Hz, 1H, CH_2_CO), 2.20 (s, 3H, CH_3_); ^13^C-NMR: 172.14, 170.09, 162.00 (3C, CO), 156.99, 134.48, 127.79 (3C, C_Ar_), 129.80 (CH_Ar)_, 129.26 (2C, CH_Ar_), 127.04 (2C, CH_Ar_), 126.81, 124.49, 120.77, 111.08 (4C, CH_Ar_), 150.45, 108.38 (2C, C_pyrazoline_), 58.81 (CH), 55.59 (OCH_3_), 39.75 (CH_2_N), 35.90, 12.12 (2C, CH_3_), 33.36 (CH_2_CO), 32.63 (CH_2_S); HRMS(EI-MS): *m/z* Calcd for C_24_H_27_N_4_O_4_S: 467.1747 [M+H]^+^, Found:467.1748 [M+H]^+^.

*3-[2-(3-Methoxyphenyl)-4-oxo-thiazolidin-3-yl]-N-(2,3-dimethyl-1-phenyl-5-oxo-pyrazolin-4-yl) propionamide* (**7f**). Yield: 75%, yellow solid; m.p. 74–76 °C; IR (ATR diamond, cm^−1^): 3247 (NH), 2930 (CH), 1667 (CONH), 1659 (CO_thiazolidine-4-one_), 1640 (CO_pyrazolin-5-one_), 641 (C-S); ^1^H-NMR: 9.33 (d, *J* = 17.4 Hz, 1H, NH), 7.45 (t, *J* = 7.7 Hz, 2H, Ar-H), 7.38 (d, *J* = 7.7 Hz, 2H, Ar-H), 7.29 (t, *J* = 7.2 Hz, 1H, Ar-H), 7.23 (dd, *J* = 11.5, 4.6 Hz, 1H, Ar-H), 6.86–6.78 (m, 3H, Ar-H), 5.82 (s, 1H, CH), 3.95–3.86 (m, 1H, CH_2_N), 3.82–3.74 (m; 3H, OCH_3_; 1H, CH_2_S), 3.66 (d, *J* = 15.4 Hz, 1H, CH_2_S), 3.09 (s, 3H, CH_3_N), 2.99–2.89 (m, 1H, CH_2_N), 2.59–2.50 (m, 1H, CH_2_CO), 2.47–2.38 (m, 1H, CH_2_CO), 2.18 (s, 3H, CH_3_); ^13^C-NMR: 171.38, 170.28, 162.09 (3C, CO), 160.10, 141.45, 134.37 (3C, C_Ar_), 130.06, 124.65, 119.20, 114.48, 112.24 (5C, CH_Ar_), 129.29 (2C, CH_Ar_), 127.19 (2C, CH_Ar_), 150.56, 108.17 (2C, C_pyrazoline_), 63.24 (CH), 55.31 (OCH_3_), 39.47 (CH_2_N), 35.78, 12.03 (2C, CH_3_), 32.95 (CH_2_CO), 32.75 (CH_2_S); HRMS (EI-MS): *m/z* Calcd for C_24_H_27_N_4_O_4_S: 467.1748 [M+H]^+^, Found: 467.1746 [M+H]^+^.

*3-[2-(4-Methoxyphenyl)-4-oxothiazolidin-3-yl]-N-(2,3-dimethyl-1-phenyl-5-oxo-pyrazolin-4-yl) propionamide* (**7g**). Yield: 86%; white solid; m.p. 120 °C; IR (ATR diamond, cm^−1^): 3247 (NH), 2929 (CH), 1656 (CONH), 1651 (CO_thiazolidine-4-one)_, 1610 (CO_pyrazolin-5-one_), 623 (C-S); ^1^H-NMR: 9.21 (s, 1H, NH), 7.45 (t, *J* = 7.7 Hz, 2H, Ar-H), 7.38 (d, *J* = 7.7 Hz, 2H, Ar-H), 7.31–7.27 (m, 1H, Ar-H), 7.19 (d, *J* = 8.6 Hz, 2H, Ar-H), 6.83 (d, *J* = 8.6 Hz, 2H, Ar-H), 5.79 (s, 1H, CH), 3.87 (dt, *J* = 13.7, 6.6 Hz, 1H, CH_2_N), 3.76 (d, *J* = 19.3 Hz; 3H, OCH_3_; 1H, CH_2_S), 3.67 (d, *J* = 15.5 Hz, 1H, CH_2_S), 3.09 (s, 3H, CH_3_N), 2.96–2.88 (m, 1H, CH_2_N), 2.53 (dt, *J* = 13.1, 6.6 Hz, 1H, CH_2_CO), 2.45-–2.36 (m, 1H, CH_2_CO), 2.18 (s, 3H, CH_3_); ^13^C-NMR: 171.30, 170.32, 162.14 (3C, CO), 160.12, 134.45, 131.46 (3C, C_Ar_), 129.37 (2C, CH_Ar_), 128.65 (2C, CH_Ar_), 127.28 (2C, CH_Ar_), 124.72, 119.20, 114.41 (3C, CH_Ar_), 150.61, 108.20 (2C, C_pyrazoline_), 63.14 (CH), 55.41 (OCH_3_), 39.33 (CH_2_N), 35.86, 12.09 (2C, CH_3_), 33.05 (CH_2_CO), 32.95 (CH_2_S); HRMS (EI-MS): *m/z* Calcd for C_24_H_27_N_4_O_4_S: 467.1747 [M+H]^+^, Found: 467.1748 [M+H]^+^.

*3-[2-(2-Nitrophenyl)-4-oxothiazolidin-3-yl]-N-(2,3-dimethyl-1-phenyl-5-oxo-pyrazolin-4-yl) propionamide* (**7h**). Yield: 62%; light yellow solid; m.p. 166 °C; IR (ATR diamond, cm^−1^): 3244 (NH), 3016 (CH), 1686 (CONH), 1647 (CO_thiazolidine-4-one_), 1622 (CO_pyrazolin-5-one_), 1522 (sym. NO_2_), 1339 (asym. NO_2_), 677 (C-S); ^1^H-NMR: 9.22 (s, 1H, NH), 8.11 (d, *J* = 8.0 Hz, 1H, Ar-H), 7.64 (t, *J* = 7.4 Hz, 1H, Ar-H), 7.47 (dd, *J* = 10.7, 4.5 Hz, 3H, Ar-H), 7.39–7.35 (m, 2H, Ar-H), 7.31 (t, *J* = 7.4 Hz, 1H, Ar-H), 7.23–7.18 (m, 1H, Ar-H), 6.35 (s, 1H, CH), 3.91–3.83 (m, 1H, CH_2_N), 3.62 (d, *J* = 15.7 Hz, 1H, CH_2_S), 3.54 (d, *J* = 15.7 Hz, 1H, CH_2_S), 3.11 (s, 3H, CH_3_N), 2.91 (dt, *J* = 13.8, 6.9 Hz, CH_2_N), 2.62 (dt, *J* = 8.0, 7.1 Hz, 1H, CH_2_CO), 2.46 (dd, *J* = 13.8, 8.0 Hz, 1H, CH_2_CO), 2.18 (s, 3H, CH_3_); ^13^C-NMR: 172.44, 170.06, 161.94 (3C, CO), 147.16, 137.34, 136.60 (3C, C_Ar_), 134.36, 125.95, 125.79 (3C, CH_Ar_), 124.81 (2C, CH_Ar_), 129.34, 128.93 (2C, CH_Ar_), 127.29 (2C, CH_Ar_), 150.47, 107.87 (2C, C_pyrazoline_), 59.25 (CH), 39.53 (CH_2_N), 35.82, 12.14 (2C, CH_3_), 33.31 (CH_2_CO), 31.40 (CH_2_S); HRMS (EI-MS): *m/z* Calcd for C_23_H_24_N_5_O_5_S: 482.1492 [M+H]^+^, Found: 482.1492 [M+H]^+^.

*3-[2-(3-Nitrophenyl)-4-oxo-thiazolidin-3-yl]-N-(2,3-dimethyl-1-phenyl-5-oxo-pyrazolin-4-yl) propionamide* (**7i**). Yield: 77%, yellow solid; m.p. 124 °C; IR (ATR diamond, cm^−1^): 3246 (NH), 2928 (CH), 1668 (CONH), 1650 (CO_thiazolidine-4-one_), 1591 (CO_pyrazolin-5-one_), 1527 (sym. NO_2_), 1349 (asym. NO_2_), 641 (C-S); ^1^H-NMR: 9.44 (s, 1H, NH), 8.16 (d, *J* = 8.3 Hz, 2H, Ar-H), 7.59 (d, *J* = 7.9 Hz, 1H, Ar-H), 7.53–7.44 (m, 3H, Ar-H), 7.40 (d, *J* = 7.9 Hz, 2H, Ar-H), 7.30 (dd, *J* = 12.6, 3.9 Hz, 1H, Ar-H), 5.99 (s, 1H, CH), 3.92–3.84 (m, 1H, CH_2_N), 3.80 (d, *J* = 15.6 Hz, 1H, CH_2_S), 3.70 (d, *J* = 15.6 Hz, 1H, CH_2_S), 3.12 (s, 3H, CH_3_N), 2.91–2.83 (m, 1H, CH_2_N), 2.59 (dt, *J* = 12.9, 6.4 Hz, 1H, CH_2_CO), 2.43 (dt, *J* = 15.6, 5.9 Hz, 1H, CH_2_CO), 2.19 (s, 3H, CH_3_); ^13^C-NMR: 171.29, 170.44, 162.05 (3C, CO), 148.65, 142.61, 134.30 (3C, C_Ar_), 133.18, 130.13, 124.90, 123.6, 122.27 (5C, CH_Ar_), 129.42 (2C, CH_Ar_), 127.46 (2C, CH_Ar_), 150.46, 107.91 (2C, C_pyrazoline_), 62.34 (CH), 39.46 (CH_2_N), 35.75, 12.06 (2C, CH_3_), 33.11 (CH_2_CO), 32.64 (CH_2_S); HRMS (EI-MS): *m/z* Calcd for C_23_H_24_N_5_O_5_S: 482.1492 [M+H]^+^, Found: 482.1493 [M+H]^+^.

*3-{2-[(3-Hydroxi-4-methoxy)phenyl]-4-oxothiazolidin-3-yl}-N-(2,3-dimethyl-1-phenyl-5-oxo-pyrazolin-4-yl) propionamide* (**7j**). Yield 62%; light yellow solid, m.p. 120–122 °C; IR (ATR diamond, cm^−1^): 3355 (OH), 3240 (NH), 2935 (CH), 1652 (CONH, CO_thiazolidine-4-one_), 1591 (CO_pyrazolin-5-one_), 667 (C-S); ^1^H-NMR: 9.03 (s, 1H, NH), 7.43 (dd, *J* = 8.4, 7.2 Hz, 2H, Ar-H, 7.35–7.32 (m, 2H, Ar-H), 7.26 (s, 1H, Ar-H), 6.88 (t, *J* = 4.3 Hz, 2H, Ar-H), 6.75 (d, *J* = 1.9 Hz, 2H, Ar-H), 5.68 (d, *J* = 1.9Hz, 1H, CH), 3.85–3.73 (m; 3H, OCH_3_; 1H, CH_2_S; 1H, CH_2_N; 1H, OH)), 3.63 (d, *J* = 15.5 Hz, 1H, CH_2_S), 3.09 (m; 3H, CH_3_N; 1H, CH_2_N), 2.54–2.38 (m, 2H, CH_2_CO), 2.16 (s, 3H, CH_3_); ^13^C-NMR: 171.46, 170.55, 162.04 (3C, CO), 147.81, 146.52, 134.32, 132.43 (4C, C_Ar_), 124.88, 118.98, 113.76, 111.16 (4C, CH_Ar_), 129.39 (2C, CH_Ar_), 127.40 (2C, CH_Ar_), 150.65, 107.83 (2C, C_pyrazoline_), 63.63 (CH), 56.02 (CH_3_O), 39.66 (CH_2_N), 35.76, 12.02 (2C, CH_3_), 33.36 (CH_2_CO), 32.95 (CH_2_S); HRMS (EI-MS): *m/z* Calcd for C_24_H_27_N_4_O_5_S: 483.1697 [M+H]^+^, Found: 483.1698 [M+H]^+^. 

*3-{2-[(3-Methoxy-4-hydroxi)phenyl]-4-oxothiazolidin-3-yl}-N-(2,3-dimethyl-1-phenyl-5-oxo-pyrazolin-4-yl) propionamide* (**7k**). Yield 67%, white solid; m.p. 123–125 °C; IR (ATR diamond, cm^−1^): 3242 (OH), 3182 (NH), 2925 (CH), 1652 (CONH, CO_thiazolidine-4-one_), 1591 (CO_pyrazolin-5-one_), 715 (C-S); ^1^H-NMR: 8.96 (s, 1H, NH), 7.44 (t, *J* = 7.8 Hz, 2H, Ar-H), 7.36 (d, *J* = 7.8 Hz, 2H, Ar-H), 7.31–7.27 (m, 2H, Ar-H), 6.81 (dd, *J* = 4.8, 3.2 Hz, 2H, Ar-H), 6.76 (dd, *J* = 8.1, 1.7 Hz, 1H, Ar-H), 6.31 (s, 1H, OH), 5.76 (s, 1H, CH), 3.84–3.72 (m; 3H, OCH_3_; 1H, CH_2_S; 1H, CH_2_N), 3.68 (d, *J* = 15.4 Hz, 1H, CH_2_S), 3.08 (s, 3H, CH_3_N), 2.96 (dd, *J* = 13.8, 6.8 Hz, 1H, CH_2_N), 2.54 (dd, *J* = 14.4, 7.5 Hz, 1H, CH_2_CO), 2.40 (dd, *J* = 14.4, 7.5 Hz, 1H, CH_2_CO), 2.18 (s, 3H, CH_3_); ^13^C-NMR: 171.40, 170.30, 161.96 (3C, CO), 147.31, 146.58, 134.34, 130.72 (4C, C_Ar_), 124.65, 120.79, 114.61, 109.46 (4C, CH_Ar_), 129.29 (2C, CH_Ar_), 127.25 (2C, CH_Ar_), 150.46, 107.99 (2C, C_pyrazoline_), 63.78 (CH), 56.04 (CH_3_O), 39.37 (CH_2_N), 35.77, 12.02 (2C, CH_3_), 33.03 (CH_2_CO), 32.99 (CH_2_S); HRMS (EI-MS): *m/z* Calcd for C_24_H_27_N_4_O_5_S: 483.1697 [M+H]^+^, Found: 483.1698 [M+H]^+^.

*3-[2-(4-Methylphenyl)-4-oxo-thiazolidin-3-yl]-N-(2,3-dimethyl-1-phenyl-5-oxo-pyrazolin-4-yl) propionamide* (**7l**). Yield 66%, light yellow solid; m.p.70–72 °C; IR (ATR diamond, cm^−1^): 3247 (NH), 3024.16 (C-H), 1668 (CONH), 1652 (CO_thiazolidine-4-one_), 1591 (CO_pyrazolin-5-one_) 632 (S-C); ^1^H-NMR: 9.46 (s, 1H, NH), 7.45 (t, *J* = 7.9 Hz, 2H, Ar-H), 7.39 (d, *J* = 7.9 Hz, 2H, 2Ar-H), 7.29 (s, 1H, Ar-H), 7.12 (s, 4H, Ar-H), 5.81 (s, 1H, CH), 3.90 (dt, *J* = 13.6, 6.9 Hz, 1H, CH_2_N), 3.76 (d, *J* = 15.5 Hz, 1H, CH_2_S), 3.66 (d, *J* = 15.5 Hz, 1H, CH_2_S), 3.09 (s, 3H, CH_3_N), 2.94–2.85 (m, 1H, CH_2_N), 2.51 (m, 1H, CH_2_CO), 2.45–2.38 (m, 1H, CH_2_CO), 2.33 (s, 3H, CH_3_), 2.19 (s, 3H, CH_3_); ^13^C-NMR: 171.30, 170.31, 162.13 (3C, CO), 138.82, 136.72, 134.38, (3C, C_Ar_), 124.65 (CH_Ar_), 129.66 (2C, CH_Ar_), 129.29 (2C, CH_Ar_), 127.19 (2C, CH_Ar_), 126.96 (2C, CH_Ar_), 150.65, 108.18 (2C, C_pyrazoline_), 63.10 (CH), 39.33 (CH_2_N), 35.78, 21.20, 12.01 (3C, CH_3_), 32.91 (CH_2_CO), 32.81 (CH_2_S); HRMS (EI-MS): *m/z* Calcd fot C_24_H_27_N_4_O_3_S: 451.1798 [M+H]^+^, Found: 451.1801 [M+H]^+^.

### 3.3. Biological Evaluation

The antioxidant activity was estimated using *in vitro* tests: ferric reducing antioxidant power, phosphomolydenum reducing antioxidant power, DPPH and ABTS radical scavenging assays.

#### 3.3.1. Ferric Reducing Antioxidant Power (FRAP) Assay

The ferric reducing power of the compounds was determined according to the procedure described in the literature [24] with minor modifications. For each compound different concentrations (10, 8, 6, 4 and 2 mg/mL in DMSO) were tested. Briefly sample (250 µL), phosphate buffer (250 µL, 0.2 M, pH 6.6) and potassium ferricyanide (250 µL, 1% w/v) were mixed in a test tube and incubated at 50 °C for 20 min in a water bath and then the reaction was stopped by adding 10% (w/v) of trichloroacetic acid solution (1 mL). After that, deionised water (1 mL) and ferric chloride (0.2 mL, 0.1% w/v) were added. The final concentrations of sample in the test tubes were 1136, 909, 682, 455 and 227 µg/mL, respectively. The mixture was left at room temperature for 10 min and then the absorbance was measured at 700 nm against a blank solution (DMSO mixed with the reagents). Increased absorbance of the reaction mixture indicated increased reducing power. For each sample the effective concentration (EC_50_) was calculated by linear regression analysis and phenazone and vitamin E (α-tocopherol) were used as reference and positive control respectively. All the tests were performed in triplicate.

#### 3.3.2. Phosphomolydenum Reducing Antioxidant Power (PRAP) Assay

The antioxidant activity of tested compounds was evaluated using the phosphomolybdenum method according to the procedure described in the literature [25] with minor modifications. The assay is based on the reduction of Mo(VI) to Mo(V) by the tested compounds and subsequent formation of green phosphate/Mo(V) complex at acid pH. For each compound different concentrations (1, 0.5, 0.25, 0.125 and 0.0625 mg/mL in DMSO) were tested. The samples (300 µL) were mixed with the reagent solution (3 mL, 28 mM sodium phosphate; 4 mM ammonium molybdate; 0.6 M sulphuric acid), incubated at 95 °C for 90 min and after that cooled at room temperature. The final concentrations of sample in the test tubes were 91, 45.5, 22.7, 11.4 and 5.7 µg/mL, respectively. The absorbance of the samples was measured at 695 nm against a blank solution (DMSO mixed with the reagents). For each sample the effective concentration (EC_50_) was calculated by linear regression analysis and phenazone and vitamin E (α-tocopherol) were used as reference and positive control respectively. All tests were performed in triplicate.

#### 3.3.3. The DPPH Radical Scavenging Assay

The antioxidant activity of the tested compounds using DPPH assay was performed in reference with [26]. For each compound different concentrations (20 mg/mL, 10 mg/mL, 5 mg/mL, 2.5 mg/mL, 1.25 mg/mL, 0.625 mg/mL in DMSO) were tested. Briefly methanolic solution of DPPH (4 mL, 15 µM) was added to tested compounds (100 µL) in a test tube. The final concentrations of sample in the test tube were 488, 244, 122, 61, 30.5 and 15. 2µg/mL, respectively. The mixture was left for 30 min at room temperature, in the dark, and after that the absorbance was measured at 515 nm against a blank solution (methanol). The radical scavenging capacity was calculated according to the following equation:

Scavenging activity % = (A_control_ − A_sample_/A_control_) × 100 (2)

where A_sample_ is the absorbance of the sample after 30 min. A_control_ is the absorbance of mixture of 100 µL DMSO and 4 mL DPPH. For each compound the effective concentration (EC_50_) was calculated by linear regression analysis and phenazone and vitamin E (α-tocopherol) were used as reference and positive control respectively. All tests were carried out in triplicate.

#### 3.3.4. The ABTS Radical Scavenging Assay

The ABTS radical scavenging ability of the compounds was tested in reference with [27] with minor modifications. The ABTS^+^ radicals were activated by reacting of ABTS (2,2'-azinobis(3-ethylbenzthiazoline-6-sulphonic acid) (7 mM) with ammonium persulphate (2.45 mM) and the mixture was left at room temperature for 16 h in the dark. The ABTS^+^ radical cation solution was diluted with ethanol to obtain an absorbance value of 0.7 ± 0.02 at 734 nm. For each compound different concentrations were tested (20, 15, 10, 5, 2.5 and 1.25 mg/mL in DMSO). To sample (50 µL), ABTS solution (1950 µL) was added. The final concentrations of sample in the test tubes were 500, 375, 250, 125, 62.5 and 31.25 µg/mL, respectively. After 6 min the absorbance was measured and the radical scavenging capacity was calculated according to the following equation:

Scavenging activity % = (A_t=0_ − A_t=6min_/A_t=0_) × 100 (3)

where A_t=0_ is the absorbance before adding the sample. A_t=6 min_ is the absorbance after 6 min of reaction. For each sample the effective concentration (EC_50_) was calculated by linear regression analysisand phenazone and vitamin E (α-tocopherol) were used as reference and positive control respectively. All tests were performed in triplicate.

#### 3.3.5. Statistical Analysis

All antioxidant assays were carried out in triplicate. Data were analyzed by an analysis of variance (ANOVA) (*p* < 0.05) and were expressed as means ± SD. The EC_50_ values were calculated by linear interpolation between the values registered above and below 50% activity.

## 4. Conclusions

In this study new heterocyclic compounds that combine the thiazolidine-4-one structure with pyrazoline-5-one ones have been synthesized. The structure of the new compounds was proved using spectroscopic methods (IR, ^1^H-NMR, ^13^C-NMR, MS). The compounds were evaluated for their antioxidant activity using *in vitro* assays: ferric reducing antioxidant power, phosphomolydenum reducing antioxidant power, DPPH and ABTS radical scavenging assays. The all tested compounds **7a**–**l** showed improved antioxidant effects in reference to phenazone. The good preliminary results which support the antioxidant potential of the synthesized compounds motivate further research focused on their anti-inflammatory effects on chronic and acute inflammation models, based on implication of oxidative stress in many disorders, including inflammation.

## References

[1] Jamwal A., Javed A., Bhardwaj V. (2013). A review on pyrazole derivatives of pharmacological potential. J. Pharm. BioSci..

[2] El-Hawash S., Badawey E.-S., El-Ashmawey I. (2006). Nonsteroidal antiinflammatory agents-part 2. Antiinflammatory, analgesic and antipyretic activity of some substituted 3-pyrazolin-5-ones and 1,2,4,5,6,7-3H-hexahydroindazol-3-ones. Eur. J. Med. Chem..

[3] Le Bourdonnec B., Meulon E., Yous S., Goossens J.-F., Houssin R., Hénichart J.-P. (2000). Synthesis and pharmacological evaluation of new pyrazolidine-3,5-diones as AT_1_ angiotensin II receptor antagonists. J. Med. Chem..

[4] El-Sayed M.A., Abdel-Aziz N.I., Abdel-Aziz A.A., El-Azab A.S., ElTahir K.E. (2012). Synthesis, biological evaluation and molecular modeling study of pyrazole and pyrazoline derivatives as selective COX-2 inhibitors and anti-inflammatory agents. Bioorg. Med. Chem..

[5] Fushimi N., Fujikura H., Shiohara H., Teranishi H., Shimizu K., Yonekubo S., Ohno K., Miyagi T., Itoh F., Shibazaki T. (2012). Structure-activity relationship studies of 4-benzyl-1*H*-pyrazol-3-yl β-d-glucopyranoside derivativesas potent and selective sodium glucose co-transporter 1 (SGLT1) inhibitors with therapeutic activity on postprandial hyperglycemia. Bioorg. Med. Chem..

[6] Khalil N.A., Ahmed E.M., El-Nassan H.B., Ahme O.K., Al-Abd A.M. (2012). Synthesis and biological evaluation of novel pyrazoline derivatives as anti-inflammatory and antioxidant agents. Arch. Pharm. Res..

[7] Shamsuzzaman K.H., Mashrai A., Sherwani A., Owais M., Siddiqui N. (2013). Synthesis and anti-tumor evaluation of B-ring substituted steroidal pyrazoline derivatives. Steroids.

[8] Markovic V., Eric S., Stanojkovic T., Gligorijevic N., Arandelovic S., Todorovic N., Trifunovic S., Manojlovic N., Jelic R., Joksovic M. (2011). Antiproliferative activity and QSAR studies of a series of new 4-aminomethylidene derivatives of some pyrazol-5-ones. Bioorg. Med. Chem. Lett..

[9] Hassan S.Y. (2013). Synthesis, antibacterial and antifungal activity of some newpyrazoline and pyrazole derivatives. Molecules.

[10] Marella A., Ali M.R., Alam M.T., Saha R., Tanwar O., Akhter M., Shaquiquzzaman M., Alam M.M. (2013). Pyrazolines: A biological review. Mini Rev. Med. Chem..

[11] Mahle F., Guimaraes T., Meira A.V., Correa R., Cruz R., Cruz A.B., Nunes R.J., Cechinel-Filho V., Campos F. (2010). Synthesis and biological evaluation of N-antipyrine-4-substituted amino-3-chloromaleimide derivatives. Eur. J. Med. Chem..

[12] Mariappan G., Saha B.P., Bhuyan N.R., Bharti P.R., Kumar D. (2010). Evaluation of antioxidant potential of pyrazolone derivatives. J. Adv. Pharm. Technol. Res..

[13] Praveen R.P.N., Kabir S.N., Mohamed T. (2010). Nonsteroidal anti-inflammatory drugs (NSAIDS): Progress in small molecule drug development. Pharmaceuticals.

[14] Rostom S.A.F., El-Ashmawy I.M., Razik H.A.A.E., Badr M.H., Ashour H.M.A. (2009). Design and synthesis of some thiazolyl and thiadiazolyl derivatives of antipyrine as potential non-acidic anti-inflammatory, analgesic and antimicrobial agents. Bioorg. Med. Chem..

[15] Prasada A., Nimavat K.S., Vyas K.B. (2012). Synthesis and biological activity of thiazolidinone containing heterocyclic compound. J. Chem. Pharm. Res..

[16] Bazrak H., Demirbas A., Demirbas N., Karaoglu S.A. (2010). Cyclization of some carbothioamide derivatives containing antipyrine and triazole moieties and investigation of their antimicrobial activities. Eur.J. Med. Chem..

[17] Antre R.V., Cendilkumar C., Goli D., Andhale G.S., Oswal R.J. (2011). Microwave assisted synthesis of novel pyrazolone derivatives attached to a pyrimidine moiety and evaluation of their anti-inflammatory, analgesic and antipyretic activities. Saudi Pharm. J..

[18] Suthar S.K., Jaiswal V., Lohan S., Bansal S., Chaudhary A., Tiwari A., Alex A.T., Joesph A. (2013). Novel quinolone substituted thiazolidin-4-ones as anti-inflammatory, anticancer agents: Design, synthesis and biological screening. Eur. J. Med. Chem..

[19] Sharma S., Sharma P.K., Kumar N., Dudhe R. (2011). A review on various heterocyclic moieties and their antitubercular activity. Biomed. Pharmacother..

[20] Patel D., Kumari P., Patel N. (2012). Synthesis and biological evaluation of some thiazolidinones as antimicrobial agents. Eur. J. Med. Chem..

[21] Rawal R.K., Tripathi R., Katti S.B., Pannecouque C., de Clercq E. (2007). Design, synthesis, and evaluation of 2-aryl-3-heteroaryl-1,3-thiazolidin-4-ones as anti-HIV agents. Bioorg. Med. Chem..

[22] Shingalapur R.V., Hosamani K.M., Keri R.S., Hugar M.H. (2010). Derivatives of benzimidazole pharmacophore: Synthesis, anticonvulsant, antidiabetic and DNA cleavage studies. Eur. J. Med. Chem..

[23] Kishore A., Nampurath G.K., Mathew S.P., Zachariah R.T., Potu B.K., Rao M.S., Valiathan M., Chamallamudi M.R. (2009). Antidiabetic effect through islet cell protection in streptozotocin diabetes: A preliminary assessment of two thiazolidin-4-ones in Swiss albino mice. Chem. Biol. Interact..

[24] Sabeena Farvin K.H., Andersen L.L., Nielsen H.H., Jacobsen C., Jakobsen G., Jahansson I., Jessen F. (2014). Antioxidant activity of cod (*Gadus morhua*) protein hydrolysates: *In vitro* assays and evaluation in 5% fish oil-in-water emulsion. Food Chem..

[25] Cervellati R., Galletti P., Greco E., Cocuzza C.E., Musumeci R., Bardini L., Paolucci F., Pori M., Soldati R., Giacomini D. (2012). Monocyclic β-lactams as antibacterial agents: Facing antioxidant activity of N-methylthio-azetidinones. Eur. J. Med. Chem..

[26] Osorio M., Aravena J., Vergara A., Taborga L., Baeza E., Catalán K., González C., Carvajal M., Carrasco H., Espinoza L. (2012). Synthesis and DPPH radical scavenging activity of prenylated phenol derivatives. Molecules.

[27] Tabassum S., Kumara T.H.S., Jasinski J.P., Millikan S.P., Yathirajan H.S., Ganapathy P.S.S., Sowmya H.B.V., More S.S., Nagendrappa G., Kaur M. (2014). Synthesis, crystal structure, ABTS radical-scavenging activity, antimicrobial and docking studies of some novel quinoline derivatives. J. Mol. Struct..

[28] Kumar S., Bhat H.R., Kumawat M.K., Singh U.P. (2013). Design and one-pot synthesis of hybrid thiazolidin-4-one-1,3,5-triazines as potent antibacterial agents against human disease-causing pathogens. New J. Chem..

[29] Apotrosoaei M., Vasincu I., Constantin S., Buron F., Routier S., Profire L. (2014). Synthesis, characterization and antioxidant activity of some new thiazolidin-4-one derivatives. Rev. Med. Chir. Soc. Med. Nat. Iasi.

[30] Theodorou V., Konstantinos S., Tzakos A.G., Ragoussis V. (2007). A simple method for the alkaline hydrolysis of esters. Tetrahedron Lett..

[31] Sambarkar P.B., Patil A.C. (2012). Synthesis of amides from acid and amine using coupling reagents. J. Curr. Pharm. Res..

